# Supplementary material for: Oligodendrocytes in human induced pluripotent stem cell-derived cortical grafts remyelinate adult rat and human cortical neurons
Source: Stem Cell Reports. 2023 May 25;18(8):1643–56. doi: 10.1016/j.stemcr.2023.04.010 (PMC10444570; doi:10.1016/j.stemcr.2023.04.010)
Supplement: Document S2. Article plus supplemental information [file mmc2.pdf]

Stem Cell Reports
Article
OPEN ACCESS

Oligodendrocytes in human induced pluripotent stem cell-derived cortical grafts remyelinate adult rat and human cortical neurons

Raquel Martinez-Curiel,[1] Linda Jansson,[1] Oleg Tsupykov,[2] Natalia Avaliani,[3] Constanza Aretio-Medina,[1] Isabel Hidalgo,[4] Emanuela Monni,[3] Johan Bengzon,[5] Galyna Skibo,[2] Olle Lindvall,[1] Zaal Kokaia,[1,*] and Sara Palma-Tortosa[1]

[1]Laboratory of Stem Cells and Restorative Neurology, Lund Stem Cell Center, Lund University, 22184 Lund, Sweden
[2]Department of Cytology, Bogomoletz Institute of Physiology; Institute of Genetic and Regenerative Medicine, Strazhesko National Scientific Center of Cardiology, Clinical and Regenerative Medicine, 01024 Kyiv, Ukraine
[3]Lund Stem Cell Center, Lund University, 22184 Lund, Sweden
[4]Division of Molecular Hematology, Wallenberg Center for Molecular Medicine, Lund Stem Cell Center, Lund University, 22184 Lund, Sweden
[5]Division of Neurosurgery, Department of Clinical Sciences Lund, University Hospital, 22184 Lund, Sweden
*Correspondence: zaal.kokaia@med.lu.se
https://doi.org/10.1016/j.stemcr.2023.04.010

SUMMARY

Neuronal loss and axonal demyelination underlie long-term functional impairments in patients affected by brain disorders such as ischemic stroke. Stem cell-based approaches reconstructing and remyelinating brain neural circuitry, leading to recovery, are highly warranted. Here, we demonstrate the *in vitro* and *in vivo* production of myelinating oligodendrocytes from a human induced pluripotent stem cell (iPSC)-derived long-term neuroepithelial stem (lt-NES) cell line, which also gives rise to neurons with the capacity to integrate into stroke-injured, adult rat cortical networks. Most importantly, the generated oligodendrocytes survive and form myelin-ensheathing human axons in the host tissue after grafting onto adult human cortical organotypic cultures. This lt-NES cell line is the first human stem cell source that, after intracerebral delivery, can repair both injured neural circuitries and demyelinated axons. Our findings provide supportive evidence for the potential future use of human iPSC-derived cell lines to promote effective clinical recovery following brain injuries.

INTRODUCTION

Several disorders affecting the human brain, such as ischemic stroke, head trauma, and multiple sclerosis, lead to both neuronal loss and axonal demyelination, which underlie the functional deficits (Benedict et al., 2020; Kuhn et al., 2019; Nasrabady et al., 2018). A major cause of demyelination is the death of oligodendrocytes, i.e., the cell population producing myelin. The brain reacts to demyelination by two compensatory mechanisms: first, through the production of new myelin from oligodendrocytes in the area surrounding the lesion (Duncan et al., 2018), resulting in fewer and, in some cases, mistargeted myelin sheets (Neely et al., 2022); second, by increased endogenous oligodendrogenesis (Franklin et al., 2021). However, this response is limited and most newly generated oligodendrocytes fail to differentiate and remyelinate (Hughes et al., 2018; Marin and Carmichael, 2019).

Transplantation of remyelinating cells derived from stem cells has the potential to become a novel approach for treating myelin loss in the human brain. Oligodendrocytes can be generated from human induced pluripotent stem cells (iPSCs) or embryonic stem cells (ESCs) using different reprogramming and differentiation protocols (Ehrlich et al., 2017; Garcia-Leon et al., 2018; Shaker et al., 2021; Wang et al., 2013). Moreover, grafted oligodendrocytes produced from human pluripotent stem cells are capable of remyelinating host axons in the rodent's central nervous system. Human ESC-derived oligodendrocyte progenitor cells (OPCs), transplanted into the demyelinated mouse spinal cord and irradiated rat brain, remyelinated host-derived demyelinated axons (Nistor et al., 2005; Piao et al., 2015). Also, human iPSC-derived OPCs and isolated $O4^+$ population (containing immature and mature oligodendrocytes), grafted into mouse injured spinal cord and demyelinated corpus callosum, remyelinated axons (Ehrlich et al., 2017; Kawabata et al., 2016). Besides replacing myelinating cells, human stem cell-derived transplants can promote remyelination in animal models by other mechanisms. For example, intracerebral transplantation of human astrocytic-fated iPSC-derived progenitors increased endogenous oligodendrogenesis and remyelination via the release of growth factors in mice with white matter stroke (Llorente et al., 2021). Whether human oligodendroglial progenitors, derived from iPSCs or ESCs, can remyelinate demyelinated tissue after transplantation in the adult human brain is unknown.

Effective repair in brain disorders using cell transplantation will require the replacement of both neurons and oligodendrocytes, capable of remyelinating host and grafted neurons. Currently, little is known whether the same human stem cell source can give rise to both functional neurons and myelinating oligodendrocytes after transplantation into the adult brain (Baker et al., 2017; Nori et al., 2011). We recently showed that human iPSC-derived long-term neuroepithelial stem (lt-NES) cells, fated toward

This is an open access article under the CC BY license (http://creativecommons.org/licenses/by/4.0/).

a cortical neuronal phenotype and transplanted into the rat cortex adjacent to an ischemic lesion, gave rise to functional cortical neurons. These neurons sent projections to both hemispheres, became integrated into host neural circuitry, and reversed sensorimotor deficits (Palma-Tortosa et al., 2020; Tornero et al., 2017). We also found that 40% of cells in the transplant, a substantial number of graft-derived cells in the corpus callosum, and a few of them in the thalamus and striatum, expressed the oligodendrocyte marker SOX10. Moreover, human-derived myelin basic protein (MBP) was observed close to the transplant, providing suggestive evidence that the graft-derived $SOX10^{+}$ cells could be oligodendrocytes contributing to remyelination.

Here, we demonstrate that the human iPSC-derived lt-NES cells, primed toward a cortical neuronal phenotype, produce bona fide oligodendrocytes both *in vitro* and *in vivo*. The generated cells display the structural, molecular, and functional characteristics of human mature oligodendrocytes and myelinate lt-NES cell-derived axons in culture as well as host-derived axons after xenotransplantation into rat stroke-injured cortex and allotransplantation into human adult cortical organotypic cultures.

RESULTS

Cortically fated human lt-NES cells form myelinating oligodendrocytes in cell culture

In our previous study (Palma-Tortosa et al., 2020), we obtained some evidence for the presence not only of neurons but also of myelin-forming oligodendrocytes in the grafts after intracerebral transplantation of cortically primed human lt-NES cells in a rat stroke model. Here, we wanted to determine, first *in vitro*, whether the lt-NES cells can form genuine functional oligodendrocytes in addition to neurons. We, therefore, differentiated lt-NES cells according to our cortical priming protocol for up to 21 days in the cell culture (Tornero et al., 2013), and analyzed protein and gene expression of different oligodendrocytes and neuronal markers.

Using immunocytochemistry, we found that undifferentiated lt-NES cells (at day 0) were positive for SOX10, a marker known to be expressed in both neuroectodermal cells and oligodendrocytes, but did not express the neuroblast marker doublecortin (DCX) or the pan-oligodendrocyte marker OLIG2 (Figure S1A). While SOX10 expression decreased with time, the expression of DCX and OLIG2 started following 4 days of differentiation with a tendency to increase (Figure S1A). Expression of MBP started on day 8 in cells displaying the bipolar morphology typical of OPCs. At later time points, we observed an increase in morphological complexity (branching) of MBP-expressing cells, characteristic of pre-myelinating and myelinating oligodendrocytes (Figure S1A). Flow cytometry showed increased O4 (a marker for pre-myelinating and myelinating oligodendrocytes) expression over time (Figure S1D). Similarly, using quantitative reverse-transcription polymerase chain reaction (qRT-PCR), we observed that our cortical priming protocol gave rise to increased *DCX*, *OLIG2*, and *MBP* gene expression starting at day 8 (Figure S1B).

We then asked if the cortically primed human lt-NES cell-derived oligodendrocytes, differentiated for 21 days, can myelinate human lt-NES cell-derived axons. At this time point, immunocytochemistry demonstrated the presence of 5% OLIG2- and 3% CC1 (a marker of mature oligodendrocytes)-expressing cells in the cultures (Figures 1A and 1B). To verify that the observed CC1 expression was not due to the presence of astrocytes in the culture, we performed double staining of CC1 with the astrocytic markers GFAP and S100β. No colocalization was found (data not shown). Moreover, flow cytometry showed that 1.5% of the cells expressed O4 (Figure 1C, 1.5% ± 0.28%, n = 4). Importantly, we found MBP expression adjacent to the axonal marker Neurofilament, suggesting that the oligodendrocytes in the culture could be involved in axonal myelination (Figure 1D).

We used electron microscopy (EM) to provide further evidence for the presence of lt-NES cell-derived oligodendrocytes and their ability to myelinate human axons in the cultures. In addition to neurons (Gronning Hansen et al., 2020), we detected a cell population exhibiting the ultrastructural features of mature oligodendrocytes, i.e., irregularly shaped dark nucleus with clumped chromatin near the inner nuclear membrane (Figure 1E). The cytoplasm was electron dense and contained short cisternae of rough endoplasmic reticulum with short mitochondria. A large number of dendrites and axons was observed in the neuropil (Figure 1E). The neural processes formed axodendritic contacts with structural characteristics of asymmetric synapses (Figure 1F), suggesting that the lt-NES cell-derived neurons had established a network already at 21 days in culture. Importantly, the lt-NES cell-derived oligodendrocytes formed loose myelin sheaths around axons, which could be the initial stage of myelination (Figure 1G).

Cortically fated human lt-NES cells form oligodendrocytes and myelinate host axons after transplantation in stroke-injured rat cortex

We then explored whether intracerebrally transplanted, cortically fated lt-NES cell-derived progenitors could remyelinate axons after stroke, i.e., an injury-causing axonal demyelination (Zuo et al., 2019). Rats were subjected to cortical stroke, implanted with human lt-NES cells close to the injury after 48 h, and sacrificed 6 months later. In all the animals, the stroke-induced neuronal loss, as

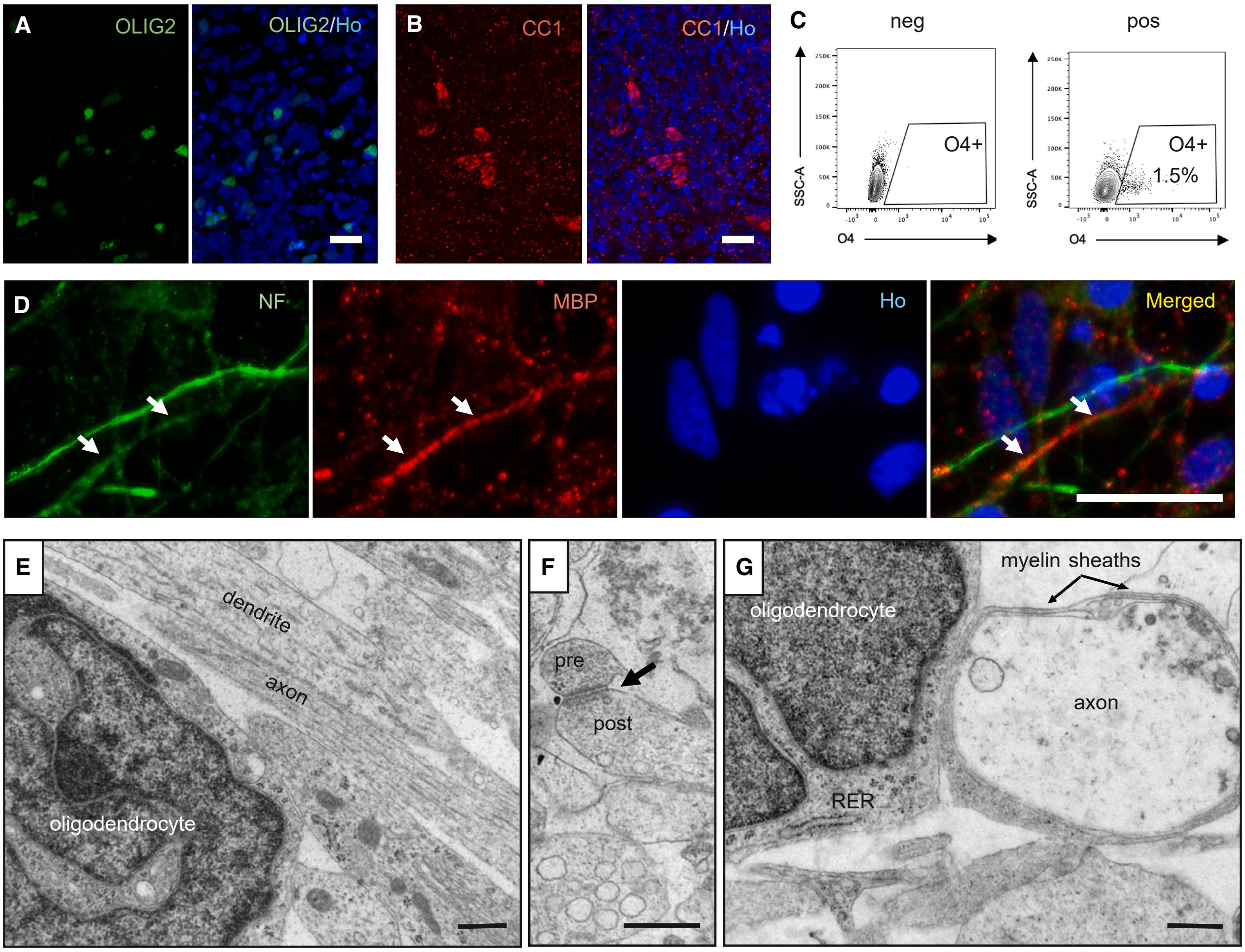

Figure 1. Cortically primed human lt-NES cells generate mature neurons and myelinating oligodendrocytes after 21 days of *in vitro* differentiation

(A and B) Confocal images of lt-NES cells differentiated for 21 days showing the expression of (A) the pan-oligodendrocyte marker OLIG2 and (B) the mature oligodendrocyte marker CC1. Representative images of n = 4 independent experiments (ind. exp.). Scale bars, 20 μm.
(C) Flow cytometry analysis of 04^+ cells, including pre-myelinating and myelinating oligodendrocytes, after 21 days of differentiation (n = 4 ind. exp.).
(D) Confocal immunohistochemical images showing axons immunoreactive for Neurofilament and myelin basic protein (MBP) expressed in closed proximity in culture (arrows depict proximity between both markers). Ho, Hoechst. Scale bars, 20 μm.
(E–G) EM images showing (E) a mature lt-NES cell-derived oligodendrocyte and neural processes, (F) an axodendritic contact with structural characteristics of the asymmetric synapse (arrow indicates a synapsis containing pre- and post-synaptic components), and (G) lt-NES cell-derived myelin loosely wrapped around an lt-NES cell-derived axon. Representative images of n = 8 ind. exp. RER, rough endoplasmic reticulum. Scale bars, 500 nm (E–G).

determined by lack of NeuN (marker of mature neurons) immunoreactivity, was restricted to the cortex (mostly somatosensory cortex [S1FF and S1BL] and motor area [M1]), sparing subcortical structures (Palma-Tortosa et al., 2020).

To study the overall distribution of axonal demyelination following stroke, we quantified the expression of MBP as a measure of myelin density (Figure 2). The ischemic insult caused decreased MBP expression in the dorsal striatum, the middle and lateral parts of the corpus callosum and its thickness and peri-infarct areas (Figures 2A–2G). Ectopic MBP expression was detected around $OLIG2^+$ cell bodies close to the injury in stroke animals (Figures 2H and 2I). Transplantation of lt-NES cells resulted in increased MBP expression in the middle part of the corpus callosum and dorsal striatum and a similar tendency in the peri-infarct area compared with non-grafted rats, whereas the thickness of corpus callosum did not

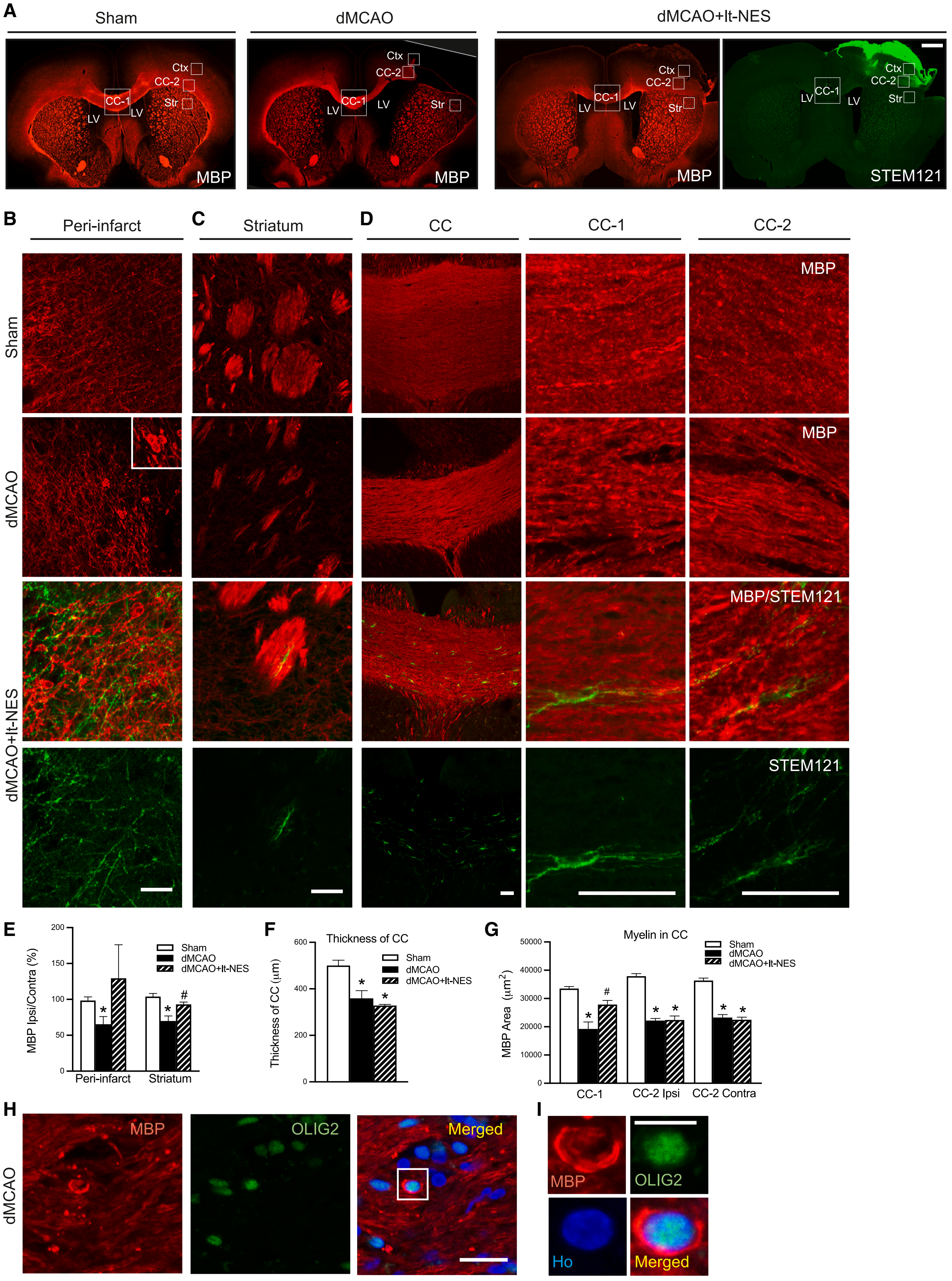

(legend on next page)

differ between the groups of stroke-affected animals (Figures 2A–2G). The presence of lt-NES cells in areas of demyelination was mainly restricted to the peri-infarct zone and corpus callosum, illustrating that the contribution of grafted cells to remyelination was limited and indicating that other myelination mechanisms were most likely triggered by the transplantation (Figures 2A–2D).

Our previous study indicated that the grafted lt-NES cell-derived neurons regulate motor behavior in stroke-injured rats through transcallosal connections to the contralateral hemisphere (Palma-Tortosa et al., 2020). We therefore analyzed in more detail the effect of stroke and lt-NES cell transplantation on the number of oligodendrocytes in the corpus callosum. Immunohistochemical analysis showed more OLIG2$^+$ cells in the middle part of the corpus callosum in animals subjected to stroke compared with sham-treated animals. In transplanted rats, the increase of OLIG2$^+$ cells was even more pronounced. The majority of OLIG2$^+$ cells did not co-express the human nuclear marker STEM101 and were most likely rat-derived cells. Some human cells were also found, even if they contributed only to a fraction of the total number of OLIG2$^+$ cells (Figures S2A–S2C).

Since we detected OLIG2$^+$ cells of human origin in the corpus callosum, we hypothesized that part of the transplanted lt-NES cell-derived progenitors had become oligodendrocytes and contributed to the remyelination. Arguing for the formation of mature oligodendrocytes *in vivo*, we found that, at 6 months after transplantation, 15%–20% of the STEM101$^+$ cells in the core of the graft expressed OLIG2 (Figure 3A) and about 20% expressed CNPase (marker for pre-myelinating and myelinating oligodendrocytes) (Figure 3C). We also observed human-derived cells expressing the mature oligodendrocyte marker, CC1, with varying density through the graft (Figure 3D). Hardly any grafted cells expressed neuron-glial antigen 2 (Figure 3B), a marker for OPCs.

To provide further evidence for the formation of mature myelinating oligodendrocytes, we performed the ultrastructural analysis 6 months after transplantation in stroke-affected animals with grafts of GFP-labeled, cortically fated human lt-NES cells. Following the validation of GFP expression in myelin sheets in our cell line (data not shown), immunoperoxidase reaction or immunogold staining using anti-GFP antibodies was carried out to localize the grafted GFP$^+$ lt-NES cells and their processes, which were easily identified due to the brown DAB reaction product in cytoplasm and processes (Figures 4A and 4B). Most GFP$^+$ cells were located in the peri-infarct area, while some were found in the corpus callosum and contralateral cortex. Some GFP$^+$ cells exhibited the morphology of mature myelinating oligodendrocytes (Figure 4C): dark electron-dense rectangular-shape cytoplasm, heterogeneous nuclear chromatin pattern, and short and wide endoplasmic reticulum cisternae organized in the vicinity of the nucleus.

To determine if the human lt-NES cell-derived oligodendrocytes could form myelin sheaths around host axons, we performed post-embedding immunogold labeling of GFP. In support of host axonal myelination by the graft-derived human oligodendrocytes, immuno-EM (iEM) demonstrated individual or clusters of gold particles within the membranous sheets of myelin (i.e., graft-derived myelin) surrounding host unlabeled axons (Figure 4D).

Grafted human lt-NES cell-derived oligodendrocytes myelinate host axons in adult human cortical tissue

We have previously shown that cortically fated lt-NES cell-derived progenitors differentiate to cortical neurons and establish functional connections with host neurons after transplantation onto organotypic cultures of adult human cortex obtained from epileptic patients undergoing surgery (Gronning Hansen et al., 2020). Here, we used this model to determine whether grafted lt-NES cells also give rise to mature oligodendrocytes that can myelinate axons in the adult human cortical environment.

We first assessed the preservation of the cortical tissue during the organotypic culture. In accordance with our previous findings (Gronning Hansen et al., 2020; Palma-Tortosa et al., 2020), we found that the expression of the neuronal marker NeuN was visually unaffected after 4 weeks in culture compared with acute tissue, whereas

Figure 2. Intracortical transplantation of human lt-NES cell-derived progenitors improves myelination in stroke-injured rat brains
(A) Coronal overview of MBP$^+$ area in the rat brain of sham-treated animals (Sham) and in animals subjected only to stroke (dMCAO) or stroke followed by transplantation (dMCAO+lt-NES). An overview of the area positive for the human cell cytoplasmic marker STEM121$^+$ is also included in the transplanted group. White squares show the areas where images were taken and quantification has been performed. Scale bar, 1 mm. CC-1, middle part of the corpus callosum; CC-2, ipsilateral part of the corpus callosum; Ctx, cortex; LV, lateral ventricle; Str: striatum.
(B–G) Confocal immunohistochemical images and quantification of MBP$^+$ area in (B and E) peri-infarct area, (C and E) dorsal striatum, and (D, F, and G) middle and lateral (ipsilateral and contralateral) parts of the corpus callosum of Sham, dMCAO, or dMCAO+lt-NES. STEM121 staining shows the contribution of graft-derived cells in different areas. Scale bars, 100 μm (B–D). n = 4–5 animals per group.
(H and I) Image showing myelin surrounding OLIG2$^+$ cell (higher magnification in H). Nuclear staining (Hoechst, blue) is included in the merged panel. Scale bars, 20 μm. *$p < 0.05$ vs. Sham. #$p < 0.05$ vs. dMCAO. Data are shown as mean ± SEM.

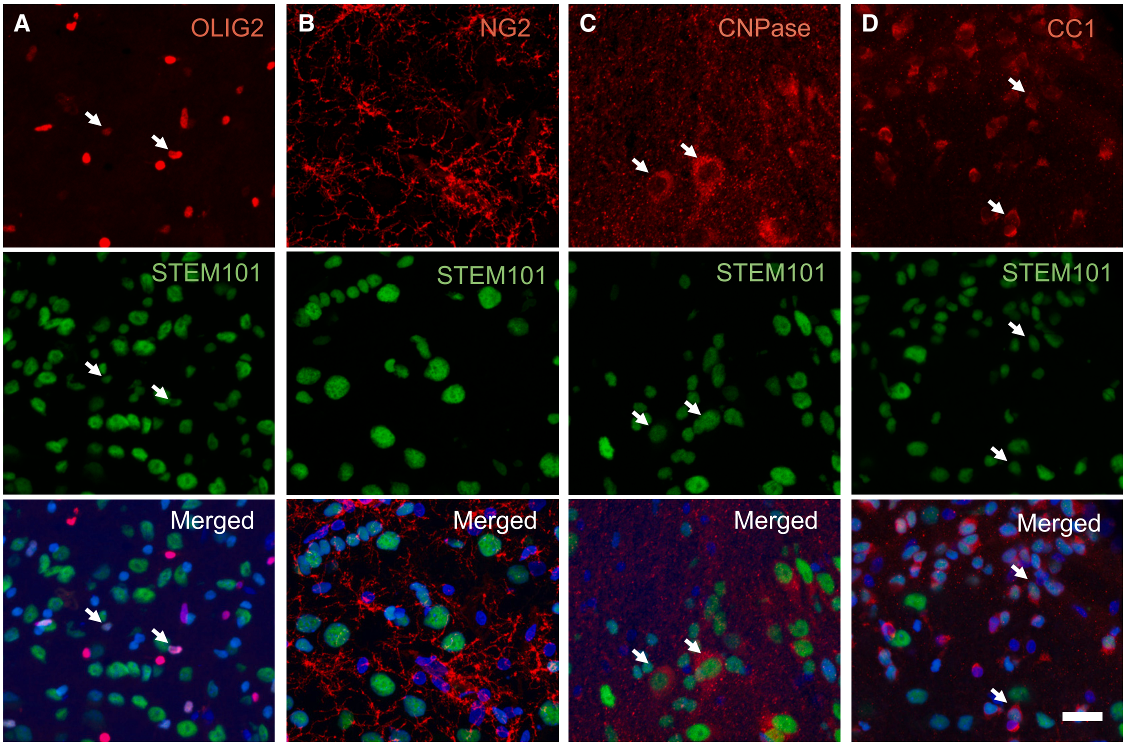

Figure 3. Cortically primed human lt-NES cell-derived progenitors give rise to mature oligodendrocytes 6 months after intracortical transplantation into stroke-injured rat somatosensory cortex

Representative confocal images of the transplantation area showing the expression of (A) the pan-oligodendrocyte marker OLIG2, (B) OPC marker neuron-glial antigen 2 (NG2), (C) marker for immature and mature oligodendrocytes CNPase, and (D) mature oligodendrocyte marker CC1. Arrows indicate the colocalization of respective markers with STEM101, a human nuclear marker. Nuclear staining (Hoechst, blue) is included in the merged panel. Scale bar, 20 μm.

MAP2 expression had decreased by more than 90% (Figures 5A and 5B). OLIG2, CNPase, and CC1 were expressed in acute tissue (Figures S3A and S3B), and MBP expression resembled the typical aligned axonal distribution in the adult human cortex (Figure S3A). After 4 weeks in culture, the expression of OLIG2, CNPase, and CC1 was unaffected (Figures S4A–S4C). Overall MBP expression was diminished compared with acute tissue (Figure S3A).

Electrophysiological analysis showed that two out of seven recorded neurons were able to fire a single action potential (AP) (Figures 5G–5I), while the majority were unable to generate any (five out of seven) (Figures 5C–5E). Inability to fire AP corresponded with small inward sodium currents, indicating that sodium conductance, which is essential for normal neuronal function and an initial phase of AP, was compromised (Figures 5F and 5J). However, passive membrane properties, such as membrane resistance (301 ± 67 MΩ) and resting membrane potential (−74.7 ± 2.5 mV), were still comparable with the values reported in fresh adult human cortical tissue (Avaliani et al., 2014), indicating that the neurons are still alive and relatively viable, although not very active.

Taken together, our data indicate that the architecture of the adult human cortical tissue is largely preserved but the functionality of the neuronal network is decreased after 4 weeks in culture.

To assess their capacity to differentiate into myelinating oligodendrocytes in the adult human cortical environment, cortically fated GFP$^+$ lt-NES cells were then transplanted onto the organotypic slice cultures (Figure S5). We found graft-derived (GFP$^+$ cells) with extensive arborizations throughout the organotypic culture after 4 weeks (Figure 6A). About 40%–50% of the GFP$^+$ cells colocalized with the pan-oligodendrocyte marker OLIG2 (Figures 6B and 6C). Importantly, colocalization of GFP$^+$ cells with either CNPase or CC1 were also found, arguing for the presence of both immature and mature graft-derived oligodendrocytes (Figures 6D–6G). Due to the cytoplasmic nature of CNPase and CC1 staining and, since a human cell marker cannot be used to identify human cells transplanted into human tissue, the percentage of grafted cells positive for these markers could not be evaluated.

We finally determined if the grafted human GFP$^+$ lt-NES cell-derived oligodendrocytes had formed myelin sheaths.

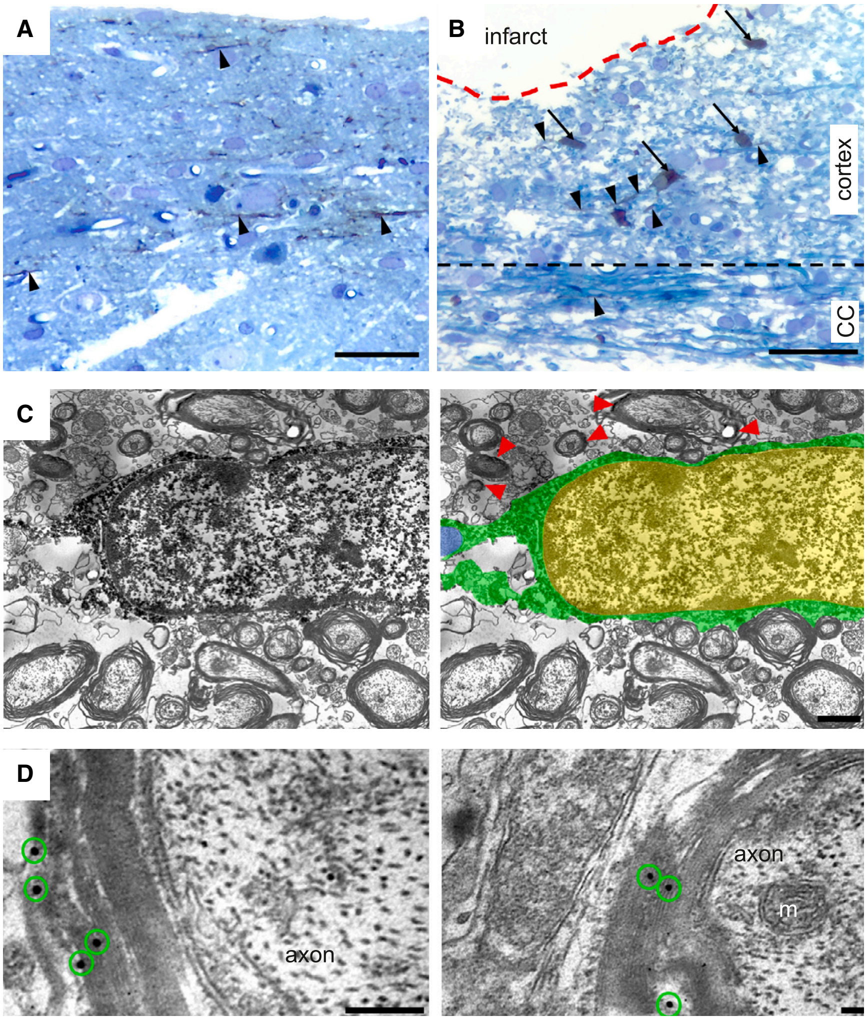

Figure 4. Grafted human lt-NES cell-derived oligodendrocytes myelinate axons in the rat injured cortex
(A and B) Light micrographs of a toluidine blue-stained plastic-embedded section from the cortex of stroke-subjected rats contralateral (A) and ipsilateral (B) to the lesion. GFP/DAB-positive lt-NES cell-derived cells were visualized in the peri-infarct area (ipsilateral cortex), corpus callosum, and contralateral cortex. Arrows highlight GFP/DAB-positive cells (brown color), and arrowheads indicate GFP/DAB-positive processes (brown color).
(C) Representative iEM image showing that, at 6 months after transplantation, GFP/DAB-positive cells in the corpus callosum exhibit the morphology of mature myelinating oligodendrocytes (for better visualization in the EM images, GFP/DAB-positive oligodendrocyte cytoplasm and processes are colored green and the nucleus is yellow). The host axon myelinated by the GFP/DAB-positive oligodendrocyte is colored blue. Red arrowheads indicate areas of GFP/DAB-positive myelin.
(D) Electron micrographs of GFP^+ immunogold particles (green circles) associated with compact myelin sheaths. CC, corpus callosum; m, mitochondrion. Scale bars, 50 μm (A and B), 2 μm (C), and 0.1 μm (D).

Anti-GFP antibodies and immunogold labeling combined with iEM demonstrated the presence of individual or clusters of gold particles within the membranous sheets of myelin (signifying graft-derived myelin) surrounding non-GFP-labeled host axons (Figures 7A and 7B). These results provide strong evidence that lt-NES cell-derived oligodendrocytes can myelinate human-derived axons after transplantation into adult human cortical tissue.

DISCUSSION

Here, we describe the *in vitro* and *in vivo* production of myelinating oligodendrocytes from a human iPSC-derived cell line, which also gives rise to neurons with the capacity to integrate into the adult human neural circuitry (Palma-Tortosa et al., 2020). Most importantly, the generated oligodendrocytes survive and form myelin-ensheathing human axons in the host tissue after grafting onto adult human cortical organotypic cultures. We have previously shown that the human iPSC-derived cortical neurons establish connections with the human host neurons in these slice cultures (Gronning Hansen et al., 2020). Taken together, we report for the first time a human iPSC-derived cell line, primed toward a cortical neuronal phenotype, with the capacity to generate both neurons and oligodendrocytes reconstructing neural circuitry and myelinating axons in the adult human cerebral cortex.

Our conclusion that the generated cells are bona fide functional oligodendrocytes is inferred from the following findings: first, the lt-NES cell-derived progenitors expressed oligodendrocyte lineage markers, such as OLIG2, O4, CNPase, and CC1, as evidenced by immunocytochemistry and flow cytometry; second, EM showed the presence of mature oligodendrocytes and myelin around lt-NES cell-derived axons after 21 days of *in vitro* differentiation; third, 6 months after intracortical transplantation into stroke-injured rat cortex, 20% of grafted cells had become

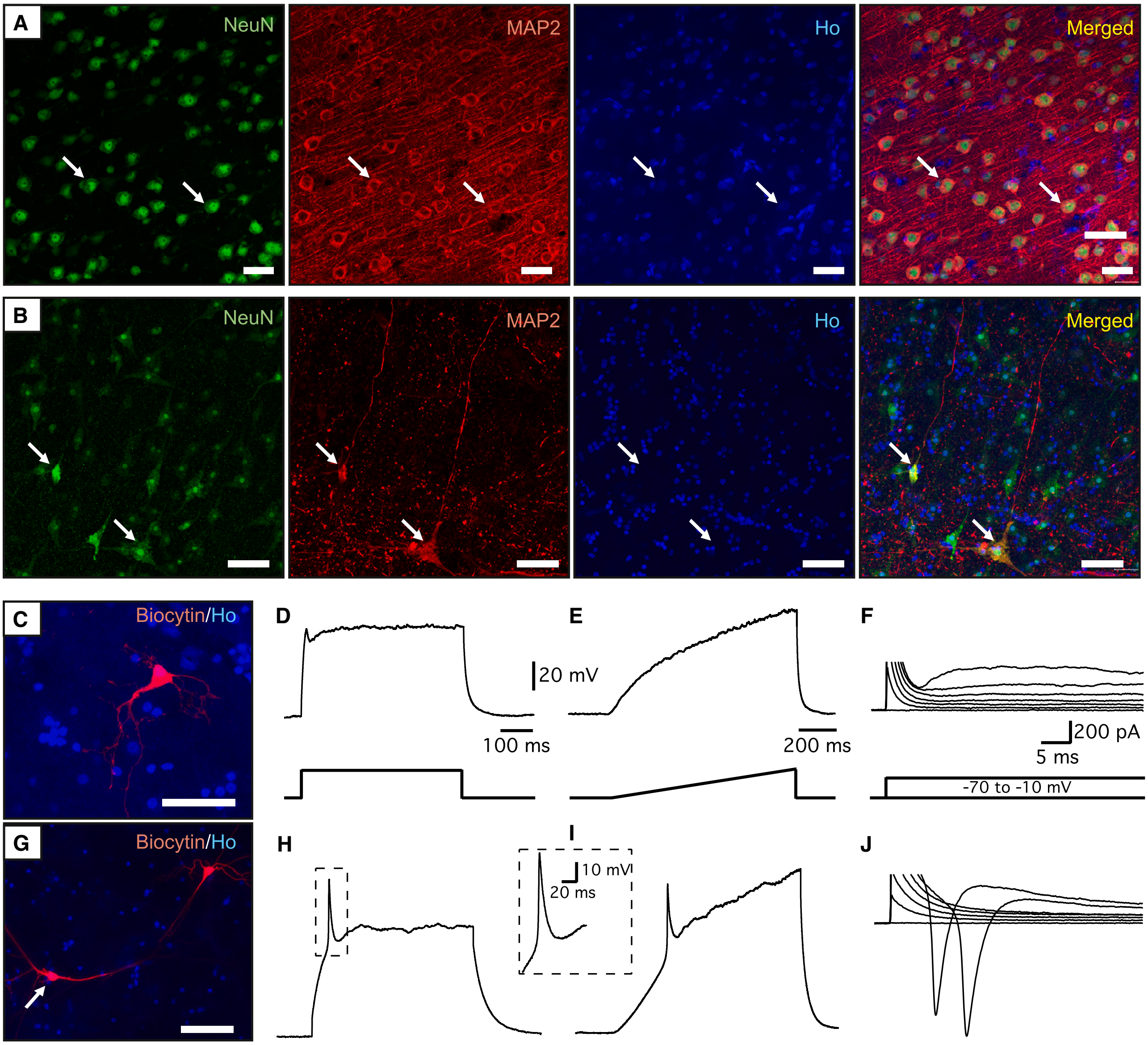

Figure 5. The functionality of neuronal circuitry is compromised in human adult cortical organotypic tissue after 4 weeks in culture

(A and B) Representative confocal images of (A) acute and (B) 4-week-cultured human cortical tissue showing the expression of the neuronal markers NeuN and MAP2. Arrows indicate colocalization. Nuclear staining (Hoechst, blue) is included in the merged panel.
(C–J) Examples of cortical neurons recorded in 4-week-cultured human cortical tissue. (C and G) Biocytin labeling of the recorded neurons. (D–F) Whole-cell patch-clamp recording traces from the cell in (C). The cell is not able to fire AP either at step (250 pA) (D) or at the ramp (0–300 pA) (E) current injection in current clamp mode. (F) The same cell has a very small inward sodium current upon 10 mV depolarization step in voltage-clamp mode at −70 mV holding potential. (H–J) Patch-clamp recording of another cell, shown in (G), that was able to generate a single AP in the similar protocol as above (H and I), with a bigger sodium current in voltage-clamp mode (J). Scale bars are the same for the upper and lower panels of the traces unless otherwise indicated. Ho, Hoechst. Scale bars, 50 μm.

oligodendrocytes, expressing OLIG2, CNPase, and CC1, and graft-derived myelin was present in areas of demyelination, as observed with MBP immunohistochemistry and EM; fourth, 1 month after transplantation onto adult human cortical cultures, grafted cells expressed markers for mature oligodendrocytes (CNPase and CC1), and graft-derived myelin was found around host human axons using immunohistochemistry and EM.

Generally, human neural progenitor cell- or stem cell-derived OPCs are used to generate myelinating

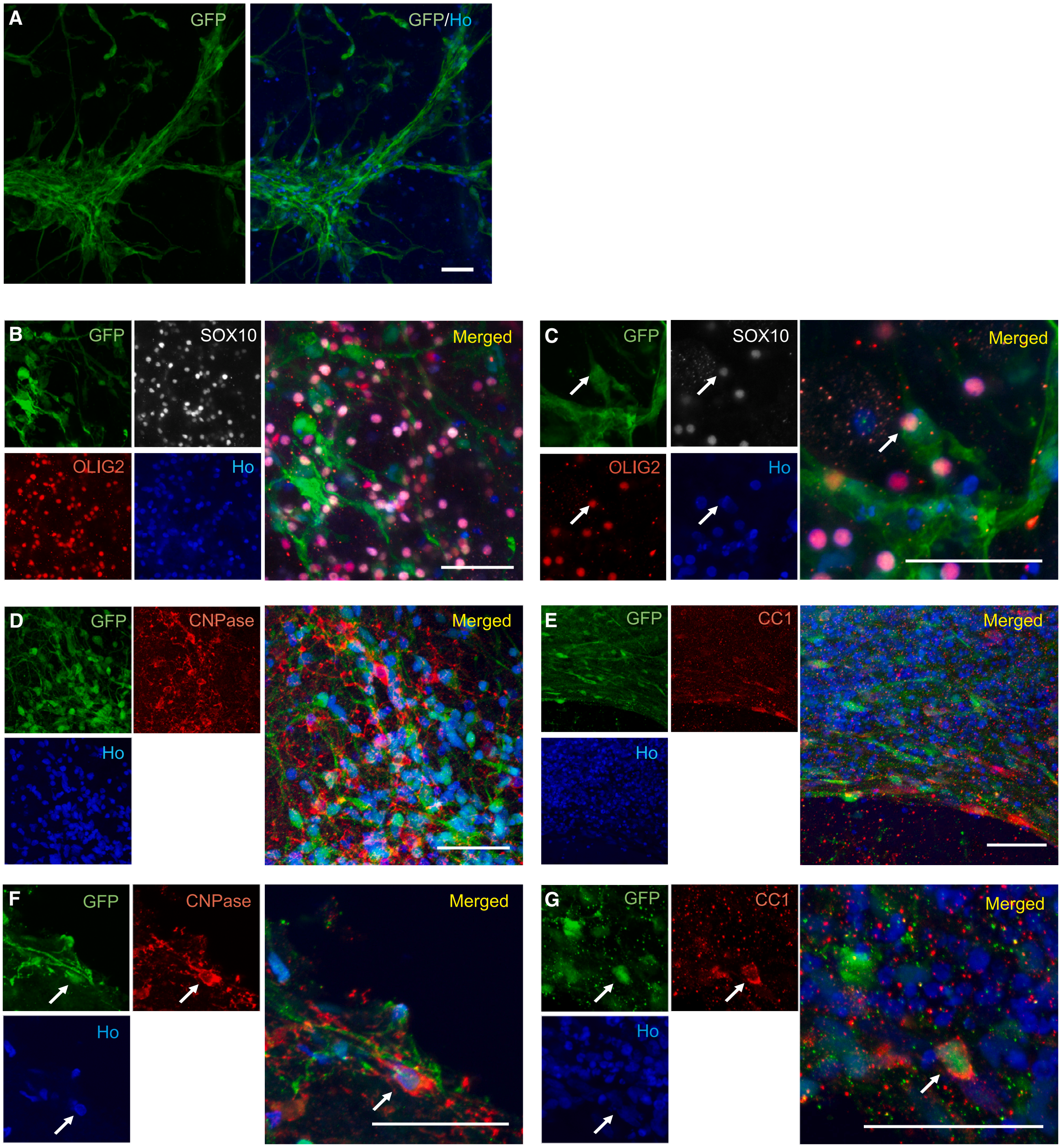

Figure 6. Human lt-NES cell-derived progenitors survive long-term and become mature oligodendrocytes 4 weeks after *ex vivo* transplantation into organotypic cultures of the human adult cortex

(A) Overview of grafted GFP$^+$ lt-NES cells 4 weeks after transplantation into the human cortical culture.

(B–G) Confocal images showing GFP$^+$ lt-NES cells after *ex vivo* transplantation expressing (B and C) SOX10 and OLIG2, (D and F) CNPase, and (E and G) CC1. (B, D, and E) Low-magnification images. (C, F, and G) High-magnification images. Ho, Hoechst. Arrows indicate colocalization. Scale bars, 50 µm.

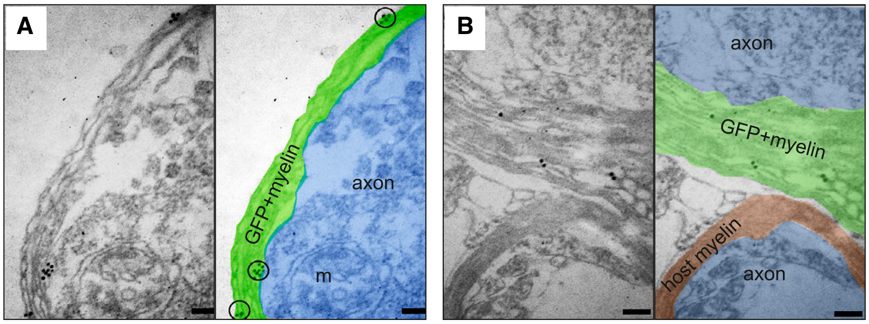

Figure 7. Human lt-NES cell-derived oligodendrocytes myelinate human-derived axons 4 weeks after *ex vivo* transplantation into organotypic cultures of the human adult cortex

(A and B) iEM images showing human grafted GFP$^+$ lt-NES cell-derived myelin wrapping human axons in organotypic cultures. For better visualization in the EM images, GFP/gold-positive myelin is colored green, GFP-negative myelin is colored red, and axons are colored blue. Black circles depict immunogold particles associated with compact myelin sheaths. m, mitochondrion. Scale bars, 0.1 μm.

oligodendrocytes (Chanoumidou et al., 2020) and neural differentiation protocols to produce functional neurons (Tornero et al., 2013; Zhao et al., 2020). Rapid concomitant generation of both mature neurons and myelinating oligodendrocytes from human stem cells in culture is a challenge. For example, differentiation of human iPSC-derived lt-NES cells into both neuronal and glial lineages gave rise to mature neurons but, even if oligodendrocytic markers were expressed, no oligodendrocyte-like morphology or axonal myelination was identified after 4 weeks (Isoda et al., 2016). Oligodendrocyte generation in a neuronally committed culture can be improved by the addition of factors that promote glial proliferation and differentiation. Thus, Ehrlich et al. (2017) reported that overexpression of SOX10, OLIG2, and NKX6.2 in human iPSC-derived neural progenitors, combined with platelet-derived growth factor (PDGF-AA), smoothened agonist, and thyroid hormone (T3), accelerates oligodendroglial differentiation, reaching 70% oligodendrocytes and 20% neurons after 28 days *in vitro*. Importantly, MBP expression was observed at day 35 of the differentiation (Ehrlich et al., 2017). Shaker et al. (2021) have shown that the generation of organoids from human neuroectodermal cells in the presence of T3, neurotrophin 3, hepatocyte growth factor, insulin growth factor, PDGF-AA, and cAMP gives rise to 29% neurons, 20% immature oligodendrocytes, and 40% myelinating oligodendrocytes at day 42 of differentiation. We found here a relatively low percentage of oligodendrocytes (5%) using our cortical differentiation protocol *in vitro*. However, functional myelination was observed already after 21 days of differentiation, i.e., earlier as compared with previous protocols (Ehrlich et al., 2017; Shaker et al., 2021). It seems possible that, also in our cultures, increased numbers of oligodendrocytes could be obtained using some of the factors mentioned above.

Previous studies with human pluripotent stem cells have reported the production of either neurons or oligodendrocytes after transplantation. In grafts of human iPSC-derived O4$^+$ cells in the spinal cord of demyelinating mice, around 80% of the cells were oligodendrocytes and only 2% expressed neuronal markers (Ehrlich et al., 2017). Similarly, human ESC-derived OPCs, transplanted in spinal cord injured rats gave rise to 94% oligodendrocytes (Sharp et al., 2010). Conversely, other intracerebral ESC- or iPSC-derived grafts have mainly contained neurons. At 60 weeks after transplantation of human ESC-derived lt-NES cells in mouse dentate gyrus and motor cortex, about 80% of cells in the grafts were neurons and no oligodendrocytes were found (Steinbeck et al., 2012). Likewise, transplantation of iPSC-derived lt-NES cells in spinal cord injured mice gave rise to 75% mature neurons and less than 1% oligodendrocytes after 7 weeks (Fujimoto et al., 2012).

Whether substantial numbers of both functional neurons and myelinating oligodendrocytes can be produced from the same intracerebral human stem cell grafts is not well studied. Nori et al. (2011) found a majority of neurons and 9% oligodendrocytes in grafts of human iPSC-derived neurospheres at 112 days after transplantation in spinal cord injured mice. Intracortical transplantation of human iPSC-derived neural stem cells in a pig model of cortical stroke gave rise to 75% neurons and 25% oligodendrocytes (Baker et al., 2017). These percentages are consistent with our observations of 40% mature neurons and 20% premyelinating and myelinating oligodendrocytes after intracortical transplantation of cortically primed lt-NES cells into stroke-injured rat cortex. Compared with the studies of Nori et al. (2011) and Baker et al. (2017), we show here for the first time that grafted human pluripotent stem *cell*-derived oligodendrocytes can be functional and myelinate axons. Most importantly, we demonstrate the survival of a large number of these oligodendrocytes and their capacity for myelination also after transplantation in an *ex vivo* model of adult human cerebral cortex. It should be pointed out, though, that our study was only designed to show proof-of-principle that grafted human

iPSC-derived oligodendrocytes can myelinate human axons. Further analysis needs to be done to determine the extent of this myelination in the human system.

We found increased endogenous oligodendrogenesis in response to stroke, as reported previously (Hernandez et al., 2021). Transplantation of human iPSC-derived progenitors further enhanced endogenous oligodendrogenesis and increased axonal remyelination in several demyelinated brain areas. Such potentiation of oligodendrogenesis, attributed to a so-called bystander effect, has been observed also following administration of other human stem cell types, e.g., after intracerebral transplantation of human iPSC-derived immature astroglia in a mouse model of neonatal white matter injury (Jiang et al., 2016) or intravenous delivery of human adipose-derived stem cells in a rat demyelinated model (Bakhtiari et al., 2021). Only a minor portion of the new oligodendrocytes originated from the grafted, cortically primed human lt-NES cells. This finding indicates that the migration of graft-derived progenitors from the transplant core to demyelinated areas and the capacity to become myelinating oligodendrocytes need to be increased to optimize functional recovery. It is important to point out, though, that the low contribution to remyelination by the grafted human-derived oligodendrocytes and the dominant role of endogenous oligodendrogenesis observed here in the rat stroke model may not reflect the outcome in a hypothetical clinical setting. Since endogenous oligodendrogenesis and remyelination efficiency is decreased by aging (Segel et al., 2019; Shen et al., 2008), replacement by grafted oligodendrocytes would probably be more critical for remyelination in older patients, who are most often affected by stroke.

Current stem cell-based approaches, which have reached patient application in stroke, aim at stimulating plasticity and dampening inflammation (de Celis-Ruiz et al., 2021; Gong et al., 2021; Hess et al., 2017), but only minor or no improvements have been so far been observed. From a clinical perspective, a human cell source that, after intracerebral delivery, has the capacity both for the functional repair of injured neural circuitries and for remyelination could be a step forward toward effective stem cell therapy for patients not only with stroke but also other brain injuries. The present findings provide supportive evidence that the further development of such a cell source is a realistic possibility.

EXPERIMENTAL PROCEDURES

Resource availability

Corresponding author

Further information and requests for resources and reagents should be directed to and will be fulfilled by the corresponding author, Zaal Kokaia (zaal.kokaia@med.lu.se).

Materials availability

This study did not generate new unique reagents.

Data and code availability

The data that support the findings of this study are available on request from the corresponding author.

Generation of iPSC-derived oligodendrocytes and cortical neurons

Human iPSC-derived lt-NES cells were produced as described previously (Falk et al., 2012; Gronning Hansen et al., 2020) (see supplemental experimental procedures). lt-NES cells used for transplantation onto *ex vivo* human tissue were transduced with a lentiviral vector carrying GFP under constitutive promoter (GFP^+ lt-NES cells). Differentiation of lt-NES cells to oligodendrocytes and neurons with a cortical phenotype was performed as described previously (Gronning Hansen et al., 2020; Tornero et al., 2013). In brief, growth factors (EGF, bFGF) and B27 were omitted and lt-NES cells were cultured at low density in a differentiation-defined medium in the presence of bone morphogenetic protein 4 (10 ng/mL, R&D Systems), wingless-type MMTV integration site family, member 3A (Wnt3A) (10 ng/mL, R&D Systems), and cyclopamine (1 μM, Calbiochem) for 7 days. On day 7, the medium was changed to Brain Phys (STEMCELL Technologies) supplemented with B27 without retinoid acid (1:50, Invitrogen) until day 21 of differentiation.

Animals and surgical procedures

All procedures were conducted following the European Union Directive (2010/63/EU) and were approved by the ethical committee for the use of laboratory animals at Lund University and the Swedish Board of Agriculture (Dnr. 5.8.18-07222/2021).

Adult athymic, nude male rats (220 g, n = 18; Charles River) were used (five sham-treated animals, five stroke-subjected animals, and eight for stroke and cell transplantation). Among transplanted animals, five were chosen for immunological characterization of the grafts and three for iEM. Details for cortical ischemic injury and cell transplantation in the somatosensory cortex are described in supplemental experimental procedures.

Organotypic cultures of the human adult cortex

Healthy human neocortical tissue was obtained with informed consent by resection of a small piece of the middle temporal gyrus from patients undergoing elective surgery for temporal lobe epilepsy (n = 3) according to guidelines approved by the Regional Ethical Committee, Lund (Dnr. 2021-07006-01). The tissue was delivered and handled as described previously (Gronning Hansen et al., 2020; Miskinyte et al., 2017). Details in the processing of the human slices are described in supplemental experimental procedures.

Transplantation of lt-NES cells onto human organotypic cortical slices

The lt-NES cell transplantation was performed as described previously (Gronning Hansen et al., 2020). In brief, GFP^+ lt-NES cells were detached at day 7 of differentiation and resuspended at a concentration of 100,000 cells/μL in pure cold Matrigel Matrix

(Corning). After part of the medium was removed from the top of the insert, the suspension mix was collected into a cold glass capillary and injected as small drops stabbing the semi-dry slice at various sites. After the Matrigel was solidified, additional medium was carefully added to completely immerse the tissue. The medium were changed once a week and co-culture was maintained for 4–6 weeks before fixation.

Immunostainings and quantifications

Details of the immunocytochemistry in cultured cells and immunohistochemistry in rat slices and human organotypic slices are described in supplemental experimental procedures. Antibodies used are included in Table S1.

Overview images of rat brain slices stained with OLIG2 and MBP were taken using a Virtual Slide Scanning System (VS-120-S6-W, Olympus, Germany).

CNPase and OLIG2 quantification in the core of the transplant in rat slices was performed in 10 μm thick 40× confocal images (LSM 780, Zeiss, Germany) and positive cells were counted by sampling different areas (($CNPase^{+}$-$STEM101^{+}$)/total $STEM101^{+}$ and ($OLIG2^{+}$-$STEM101^{+}$)/total $STEM101^{+}$).

Quantification of $OLIG2^{+}$ cells in the corpus callosum in rat slices was performed in 20× confocal images (10 μm thick z stack). For analysis of myelination, confocal images (5 μm thick z stack) were taken in the corpus callosum (40×), peri-infarct area (20×), and striatum (20×). For analysis of the dorsal lateral striatum, starting 700 μm lateral to the dorsal part of the lateral ventricle, two 20× images (x = 700 and 1,400 μm) were taken. To measure the thickness of the corpus callosum, confocal images were taken with a 20× Zoom 0.6. All quantifications were performed on maximum-intensity projection images using ImageJ software by blinded researchers.

Quantification of GFP^{+} $OLIG2^{+}$ cells in the human organotypic slices was carried out using 20× confocal images in areas where transplantation was observed, and the number of double-positive cells counted through a z stack by sampling different single planes.

Flow cytometry

The lt-NES cell cultures were harvested and washed before antibody incubation. Anti-O4 APC-conjugated antibody (Miltenyi, no. 130-119-155) was diluted 1:200 in FACS buffer (PBS + 2% fetal bovine serum + 2 nM ethylenediaminetetraacetic acid). Cells were incubated for 30 min at +4°C in darkness, followed by wash and incubation with propidium iodide (Life Technologies), a viability marker, diluted 1:1,000 in FACS buffer at least 5 min before acquisition. Cells were analyzed in an LSR II flow cytometer (Becton Dickinson) and results were analyzed with BD FACSDiva 9.0 software (BD Biosciences) and FlowJo v.10.8 Software (BD Life Sciences). The gating strategy is shown in Figure S1C.

qRT-PCR

qRT-PCR was performed on RNA extracted from cells at different time points of differentiation (D0, D4, D8, D12, and D15). RNA extraction was performed with the RNeasy Mini Kit (QIAGEN) following the protocol described by the manufacturer. RNA purity and concentration of samples were determined using a NanoDrop spectrophotometer (ND-1000). RNA (1 μg) was used for cDNA synthesis with qScript cDNA SuperMix (QuantaBio). TaqMan probes (Thermo Fisher Scientific; *OLIG2*, HS00300164_s1; *MBP*, HS00921945_m1; *DCX*, HS00167057_m1; *GAPDH*, HS02786624_g1) were used and qRT-PCR was run in triplicate samples on an iQ5 real-time cycler (Bio-Rad) with GAPDH as the housekeeping gene.

EM

For *in vitro* EM, lt-NES cell cultures (n = 8 wells) were fixed with 2% formaldehyde and 0.2% glutaraldehyde in 0.1 M phosphate buffer (pH 7.4). Samples were frozen and cut into ultrathin sections with a diamond knife. Ultrathin sections were examined and photographed using a transmission electron microscope FEI Tecnai Biotwin 120 kv.

For *in vivo* iEM, 6 months after transplantation, three rats were deeply anesthetized with pentobarbital and transcardially perfused with 0.1 M PBS followed by ice-cold 2% formaldehyde, containing 0.2% glutaraldehyde, in 0.1 M PBS (pH 7.4). Brains were removed, post-fixed for 1 h and washed in 0.1 M PBS. For *ex vivo* iEM the same fixation was performed for 30 min. Both rat and human tissue were processed as described in supplemental experimental procedures.

Electrophysiological recordings

Electrophysiological recordings were performed on 4-week-old human adult cortical organotypic slices as described previously (Andersson et al., 2016). Details are described in supplemental experimental procedures.

Statistical analysis

Statistical analysis was performed using Prism 9 software (GraphPad). An unpaired t test was used when data were normally distributed, whereas a Mann-Whitney U test was used when data did not pass the normality test. When different independent groups were compared, a one-way ANOVA plus Tukey's multiple comparison tests were performed. Significance was set at $p < 0.05$. Data are mean ± SEM.

SUPPLEMENTAL INFORMATION

Supplemental information can be found online at https://doi.org/10.1016/j.stemcr.2023.04.010.

AUTHOR CONTRIBUTIONS

S.P.-T., O.L., and Z.K. conceived the project. R.M.-C., L.J., C.A.-M., N.A., O.T., I.H., E.M., and S.P.-T. conducted the experiments and analyzed the data. S.P.-T., O.L., and Z.K. wrote the manuscript. J.B. provided human material. R.M.-C., N.A., E.M., G.S., and O.T. were involved in the collection and/or assembly of data, data analysis, and interpretation. All authors reviewed and edited the manuscript.

ACKNOWLEDGMENTS

This work is supported by grants from the Swedish Research Council, Swedish Brain Foundation, Swedish Stroke Foundation, Region Skåne, Neurofonden, the Thorsten and Elsa Segerfalk Foundation,

Rut och Erik Hardebo Foundation, and the Swedish Government Initiative for Strategic Research Areas (StemTherapy).

CONFLICT OF INTERESTS

The authors declare no competing interests.

Received: December 25, 2022
Revised: April 24, 2023
Accepted: April 25, 2023
Published: May 25, 2023

REFERENCES

Andersson, M., Avaliani, N., Svensson, A., Wickham, J., Pinborg, L.H., Jespersen, B., Christiansen, S.H., Bengzon, J., Woldbye, D.P.D., and Kokaia, M. (2016). Optogenetic control of human neurons in organotypic brain cultures. Sci. Rep. *6*, 24818. https://doi.org/10.1038/srep24818.

Avaliani, N., Sørensen, A.T., Ledri, M., Bengzon, J., Koch, P., Brüstle, O., Deisseroth, K., Andersson, M., and Kokaia, M. (2014). Optogenetics reveal delayed afferent synaptogenesis on grafted human-induced pluripotent stem cell-derived neural progenitors. Stem Cell. *32*, 3088–3098. https://doi.org/10.1002/stem.1823.

Baker, E.W., Platt, S.R., Lau, V.W., Grace, H.E., Holmes, S.P., Wang, L., Duberstein, K.J., Howerth, E.W., Kinder, H.A., Stice, S.L., et al. (2017). Induced pluripotent stem cell-derived neural stem cell therapy enhances recovery in an ischemic stroke pig model. Sci. Rep. *7*, 10075. https://doi.org/10.1038/s41598-017-10406-x.

Bakhtiari, M., Ghasemi, N., Salehi, H., Amirpour, N., Kazemi, M., and Mardani, M. (2021). Evaluation of Edaravone effects on the differentiation of human adipose derived stem cells into oligodendrocyte cells in multiple sclerosis disease in rats. Life Sci. *282*, 119812. https://doi.org/10.1016/j.lfs.2021.119812.

Benedict, R.H.B., Amato, M.P., DeLuca, J., and Geurts, J.J.G. (2020). Cognitive impairment in multiple sclerosis: clinical management, MRI, and therapeutic avenues. Lancet Neurol. *19*, 860–871. https://doi.org/10.1016/S1474-4422(20)30277-5.

Chanoumidou, K., Mozafari, S., Baron-Van Evercooren, A., and Kuhlmann, T. (2020). Stem cell derived oligodendrocytes to study myelin diseases. Glia *68*, 705–720. https://doi.org/10.1002/glia.23733.

de Celis-Ruiz, E., Fuentes, B., Moniche, F., Montaner, J., Borobia, A.M., Gutiérrez-Fernández, M., and Díez-Tejedor, E. (2021). Allogeneic adipose tissue-derived mesenchymal stem cells in ischaemic stroke (AMASCIS-02): a phase IIb, multicentre, double-blind, placebo-controlled clinical trial protocol. BMJ Open *11*, e051790. https://doi.org/10.1136/bmjopen-2021-051790.

Duncan, I.D., Radcliff, A.B., Heidari, M., Kidd, G., August, B.K., and Wierenga, L.A. (2018). The adult oligodendrocyte can participate in remyelination. Proc. Natl. Acad. Sci. USA *115*, E11807–E11816. https://doi.org/10.1073/pnas.1808064115.

Ehrlich, M., Mozafari, S., Glatza, M., Starost, L., Velychko, S., Hallmann, A.L., Cui, Q.L., Schambach, A., Kim, K.P., Bachelin, C., et al. (2017). Rapid and efficient generation of oligodendrocytes from human induced pluripotent stem cells using transcription factors. Proc. Natl. Acad. Sci. USA *114*, E2243–E2252. https://doi.org/10.1073/pnas.1614412114.

Falk, A., Koch, P., Kesavan, J., Takashima, Y., Ladewig, J., Alexander, M., Wiskow, O., Tailor, J., Trotter, M., Pollard, S., et al. (2012). Capture of neuroepithelial-like stem cells from pluripotent stem cells provides a versatile system for in vitro production of human neurons. PLoS One *7*, e29597. https://doi.org/10.1371/journal.pone.0029597.

Franklin, R.J.M., Frisén, J., and Lyons, D.A. (2021). Revisiting remyelination: towards a consensus on the regeneration of CNS myelin. Semin. Cell Dev. Biol. *116*, 3–9. https://doi.org/10.1016/j.semcdb.2020.09.009.

Fujimoto, Y., Abematsu, M., Falk, A., Tsujimura, K., Sanosaka, T., Juliandi, B., Semi, K., Namihira, M., Komiya, S., Smith, A., and Nakashima, K. (2012). Treatment of a mouse model of spinal cord injury by transplantation of human induced pluripotent stem cell-derived long-term self-renewing neuroepithelial-like stem cells. Stem Cell. *30*, 1163–1173. https://doi.org/10.1002/stem.1083.

García-León, J.A., Kumar, M., Boon, R., Chau, D., One, J., Wolfs, E., Eggermont, K., Berckmans, P., Gunhanlar, N., de Vrij, F., et al. (2018). SOX10 single transcription factor-based fast and efficient generation of oligodendrocytes from human pluripotent stem cells. Stem Cell Rep. *10*, 655–672. https://doi.org/10.1016/j.stemcr.2017.12.014.

Gong, P., Zhang, W., He, Y., Wang, J., Li, S., Chen, S., Ye, Q., and Li, M. (2021). Classification and characteristics of mesenchymal stem cells and its potential therapeutic mechanisms and applications against ischemic stroke. Stem Cell. Int. *2021*, 2602871. https://doi.org/10.1155/2021/2602871.

Grønning Hansen, M., Laterza, C., Palma-Tortosa, S., Kvist, G., Monni, E., Tsupykov, O., Tornero, D., Uoshima, N., Soriano, J., Bengzon, J., et al. (2020). Grafted human pluripotent stem cell-derived cortical neurons integrate into adult human cortical neural circuitry. Stem Cells Transl. Med. *9*, 1365–1377. https://doi.org/10.1002/sctm.20-0134.

Hernandez, I.H., Villa-Gonzalez, M., Martin, G., Soto, M., and Perez-Alvarez, M.J. (2021). Glial cells as therapeutic approaches in brain ischemia-reperfusion injury. Cells *10*. https://doi.org/10.3390/cells10071639.

Hess, D.C., Wechsler, L.R., Clark, W.M., Savitz, S.I., Ford, G.A., Chiu, D., Yavagal, D.R., Uchino, K., Liebeskind, D.S., Auchus, A.P., et al. (2017). Safety and efficacy of multipotent adult progenitor cells in acute ischaemic stroke (MASTERS): a randomised, double-blind, placebo-controlled, phase 2 trial. Lancet Neurol. *16*, 360–368. https://doi.org/10.1016/S1474-4422(17)30046-7.

Hughes, E.G., Orthmann-Murphy, J.L., Langseth, A.J., and Bergles, D.E. (2018). Myelin remodeling through experience-dependent oligodendrogenesis in the adult somatosensory cortex. Nat. Neurosci. *21*, 696–706. https://doi.org/10.1038/s41593-018-0121-5.

Isoda, M., Kohyama, J., Iwanami, A., Sanosaka, T., Sugai, K., Yamaguchi, R., Matsumoto, T., Nakamura, M., and Okano, H. (2016). Robust production of human neural cells by establishing neuroepithelial-like stem cells from peripheral blood mononuclear cell-derived feeder-free iPSCs under xeno-free conditions. Neurosci. Res. *110*, 18–28. https://doi.org/10.1016/j.neures.2016.04.003.

Jiang, P., Chen, C., Liu, X.B., Pleasure, D.E., Liu, Y., and Deng, W. (2016). Human iPSC-derived immature astroglia promote oligodendrogenesis by increasing TIMP-1 secretion. Cell Rep. *15*, 1303–1315. https://doi.org/10.1016/j.celrep.2016.04.011.

Kawabata, S., Takano, M., Numasawa-Kuroiwa, Y., Itakura, G., Kobayashi, Y., Nishiyama, Y., Sugai, K., Nishimura, S., Iwai, H., Isoda, M., et al. (2016). Grafted human iPS cell-derived oligodendrocyte precursor cells contribute to robust remyelination of demyelinated axons after spinal cord injury. Stem Cell Rep. *6*, 1–8. https://doi.org/10.1016/j.stemcr.2015.11.013.

Kuhn, S., Gritti, L., Crooks, D., and Dombrowski, Y. (2019). Oligodendrocytes in development, myelin generation and beyond. Cells *8*. https://doi.org/10.3390/cells8111424.

Llorente, I.L., Xie, Y., Mazzitelli, J.A., Hatanaka, E.A., Cinkornpumin, J., Miller, D.R., Lin, Y., Lowry, W.E., and Carmichael, S.T. (2021). Patient-derived glial enriched progenitors repair functional deficits due to white matter stroke and vascular dementia in rodents. Sci. Transl. Med. *13*, eaaz6747. https://doi.org/10.1126/scitranslmed.aaz6747.

Marin, M.A., and Carmichael, S.T. (2019). Mechanisms of demyelination and remyelination in the young and aged brain following white matter stroke. Neurobiol. Dis. *126*, 5–12. https://doi.org/10.1016/j.nbd.2018.07.023.

Miskinyte, G., Devaraju, K., Grønning Hansen, M., Monni, E., Tornero, D., Woods, N.B., Bengzon, J., Ahlenius, H., Lindvall, O., and Kokaia, Z. (2017). Direct conversion of human fibroblasts to functional excitatory cortical neurons integrating into human neural networks. Stem Cell Res. Ther. *8*, 207. https://doi.org/10.1186/s13287-017-0658-3.

Nasrabady, S.E., Rizvi, B., Goldman, J.E., and Brickman, A.M. (2018). White matter changes in Alzheimer's disease: a focus on myelin and oligodendrocytes. Acta Neuropathol. Commun. *6*, 22. https://doi.org/10.1186/s40478-018-0515-3.

Neely, S.A., Williamson, J.M., Klingseisen, A., Zoupi, L., Early, J.J., Williams, A., and Lyons, D.A. (2022). New oligodendrocytes exhibit more abundant and accurate myelin regeneration than those that survive demyelination. Nat. Neurosci. *25*, 415–420. https://doi.org/10.1038/s41593-021-01009-x.

Nistor, G.I., Totoiu, M.O., Haque, N., Carpenter, M.K., and Keirstead, H.S. (2005). Human embryonic stem cells differentiate into oligodendrocytes in high purity and myelinate after spinal cord transplantation. Glia *49*, 385–396. https://doi.org/10.1002/glia.20127.

Nori, S., Okada, Y., Yasuda, A., Tsuji, O., Takahashi, Y., Kobayashi, Y., Fujiyoshi, K., Koike, M., Uchiyama, Y., Ikeda, E., et al. (2011). Grafted human-induced pluripotent stem-cell-derived neurospheres promote motor functional recovery after spinal cord injury in mice. Proc. Natl. Acad. Sci. USA *108*, 16825–16830. https://doi.org/10.1073/pnas.1108077108.

Palma-Tortosa, S., Tornero, D., Grønning Hansen, M., Monni, E., Hajy, M., Kartsivadze, S., Aktay, S., Tsupykov, O., Parmar, M., Deisseroth, K., et al. (2020). Activity in grafted human iPS cell-derived cortical neurons integrated in stroke-injured rat brain regulates motor behavior. Proc. Natl. Acad. Sci. USA *117*, 9094–9100. https://doi.org/10.1073/pnas.2000690117.

Piao, J., Major, T., Auyeung, G., Policarpio, E., Menon, J., Droms, L., Gutin, P., Uryu, K., Tchieu, J., Soulet, D., and Tabar, V. (2015). Human embryonic stem cell-derived oligodendrocyte progenitors remyelinate the brain and rescue behavioral deficits following radiation. Cell Stem Cell *16*, 198–210. https://doi.org/10.1016/j.stem.2015.01.004.

Segel, M., Neumann, B., Hill, M.F.E., Weber, I.P., Viscomi, C., Zhao, C., Young, A., Agley, C.C., Thompson, A.J., Gonzalez, G.A., et al. (2019). Niche stiffness underlies the ageing of central nervous system progenitor cells. Nature *573*, 130–134. https://doi.org/10.1038/s41586-019-1484-9.

Shaker, M.R., Pietrogrande, G., Martin, S., Lee, J.H., Sun, W., and Wolvetang, E.J. (2021). Rapid and efficient generation of myelinating human oligodendrocytes in organoids. Front. Cell. Neurosci. *15*, 631548. https://doi.org/10.3389/fncel.2021.631548.

Sharp, J., Frame, J., Siegenthaler, M., Nistor, G., and Keirstead, H.S. (2010). Human embryonic stem cell-derived oligodendrocyte progenitor cell transplants improve recovery after cervical spinal cord injury. Stem Cell. *28*, 152–163. https://doi.org/10.1002/stem.245.

Shen, S., Sandoval, J., Swiss, V.A., Li, J., Dupree, J., Franklin, R.J.M., and Casaccia-Bonnefil, P. (2008). Age-dependent epigenetic control of differentiation inhibitors is critical for remyelination efficiency. Nat. Neurosci. *11*, 1024–1034. https://doi.org/10.1038/nn.2172.

Steinbeck, J.A., Koch, P., Derouiche, A., and Brüstle, O. (2012). Human embryonic stem cell-derived neurons establish region-specific, long-range projections in the adult brain. Cell. Mol. Life Sci. *69*, 461–470. https://doi.org/10.1007/s00018-011-0759-6.

Tornero, D., Tsupykov, O., Granmo, M., Rodriguez, C., Grønning-Hansen, M., Thelin, J., Smozhanik, E., Laterza, C., Wattananit, S., Ge, R., et al. (2017). Synaptic inputs from stroke-injured brain to grafted human stem cell-derived neurons activated by sensory stimuli. Brain *140*, 692–706. https://doi.org/10.1093/brain/aww347.

Tornero, D., Wattananit, S., Grønning Madsen, M., Koch, P., Wood, J., Tatarishvili, J., Mine, Y., Ge, R., Monni, E., Devaraju, K., et al. (2013). Human induced pluripotent stem cell-derived cortical neurons integrate in stroke-injured cortex and improve functional recovery. Brain *136*, 3561–3577. https://doi.org/10.1093/brain/awt278.

Wang, S., Bates, J., Li, X., Schanz, S., Chandler-Militello, D., Levine, C., Maherali, N., Studer, L., Hochedlinger, K., Windrem, M., and Goldman, S.A. (2013). Human iPSC-derived oligodendrocyte progenitor cells can myelinate and rescue a mouse model of congenital hypomyelination. Cell Stem Cell *12*, 252–264. https://doi.org/10.1016/j.stem.2012.12.002.

Zhao, S., Duan, K., Ai, Z., Niu, B., Chen, Y., Kong, R., and Li, T. (2020). Generation of cortical neurons through large-scale expanding neuroepithelial stem cell from human pluripotent stem cells. Stem Cell Res. Ther. *11*, 431. https://doi.org/10.1186/s13287-020-01939-6.

Zuo, M., Guo, H., Wan, T., Zhao, N., Cai, H., Zha, M., Xiong, Y., Xie, Y., Ye, R., and Liu, X. (2019). Wallerian degeneration in experimental focal cortical ischemia. Brain Res. Bull. *149*, 194–202. https://doi.org/10.1016/j.brainresbull.2019.04.023.

Stem Cell Reports, Volume *18*

Supplemental Information

Oligodendrocytes in human induced pluripotent stem cell-derived cortical grafts remyelinate adult rat and human cortical neurons

Raquel Martinez-Curiel, Linda Jansson, Oleg Tsupykov, Natalia Avaliani, Constanza Aretio-Medina, Isabel Hidalgo, Emanuela Monni, Johan Bengzon, Galyna Skibo, Olle Lindvall, Zaal Kokaia, and Sara Palma-Tortosa

SUPPLEMENTAL FIGURES AND TABLES

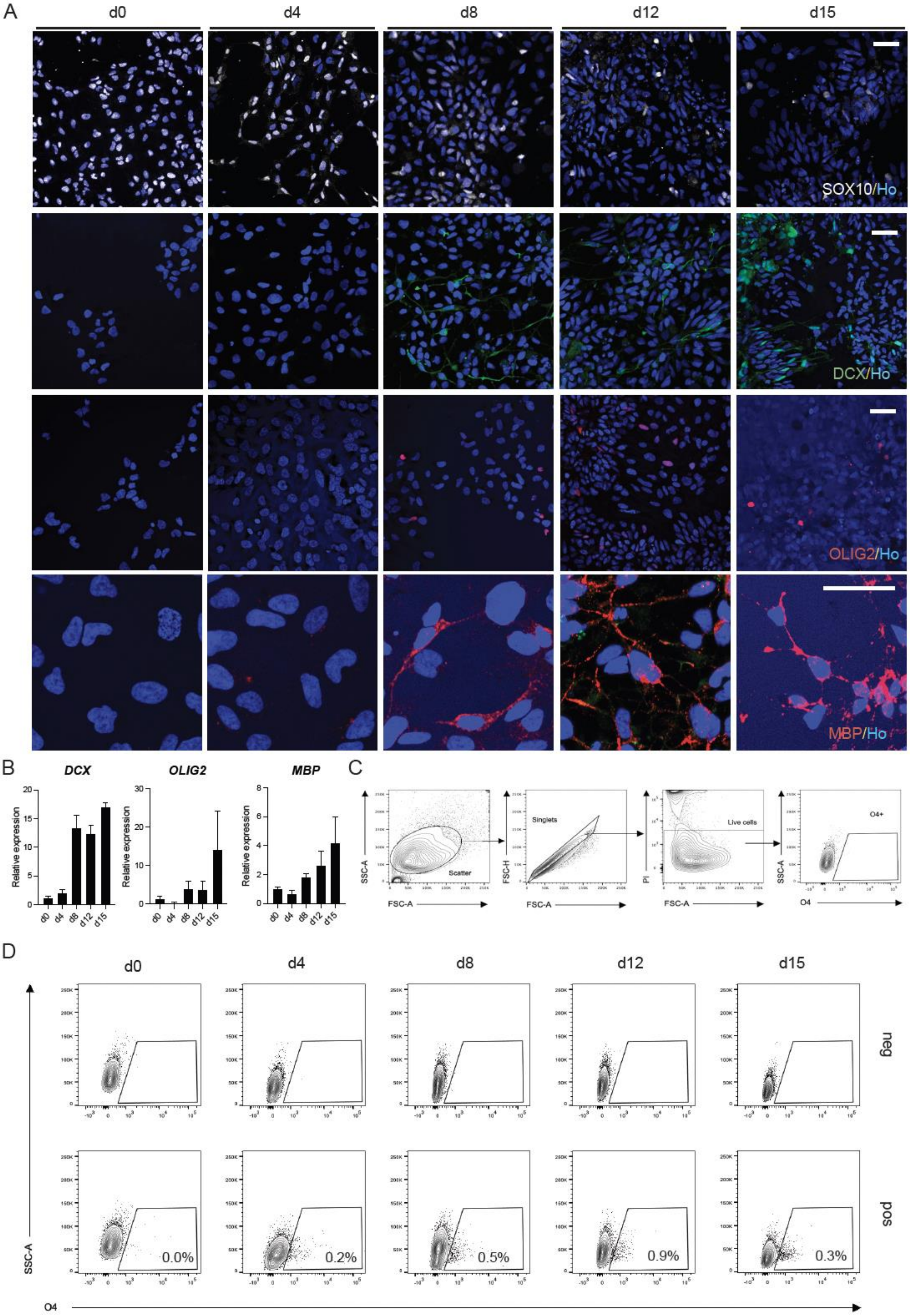

Figure S1. *In vitro* differentiation of cortically-primed human lt-NES cells gives rise to a concomitant generation of neurons and oligodendrocytes. Related to section: *Cortically fated human lt-NES cells form myelinating oligodendrocytes in cell culture*. (**A**) Confocal images of lt-NES cells at different time points of differentiation showing expression of the neuroectodermal and oligodendrocyte marker SOX10, the neuronal progenitor marker DCX, the pan-oligodendrocyte marker OLIG2, and myelin basic protein MBP (Representative images of n=4 independent experiments (ind. exp.)). (**B**) *DCX*, *OLI2*, and *MBP* changes in gene expression using RT-qPCR (n=3-4 ind. exp. per time point). (**C**) Gating strategy used for flow cytometry analysis. (**D**) Flow cytometry analysis of live cells for $O4^+$ cells at 0, 4, 8, 12, and 15 days of differentiation. Negative control (without antibody) is shown on the top of the panel. Scale bar, 20 mm. Ho, Hoechst. Data are shown as mean ± SEM.

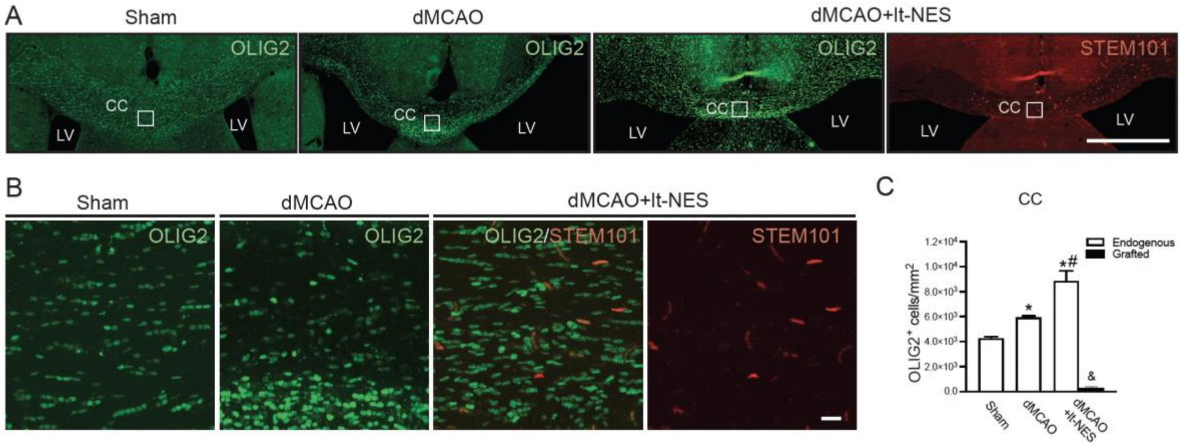

Figure S2. Intracortical transplantation of human lt-NES cell-derived progenitors increases endogenous oligodendrogenesis in stroke-injured rat brains. Related to section: *Cortically fated human lt-NES cells form oligodendrocytes and myelinate host axons after transplantation in stroke-injured rat cortex.* (**A**) Overview of OLIG2$^+$ cells in the rat corpus callosum of sham-treated animals (Sham), and in animals subjected only to stroke (dMCAO) or stroke followed by transplantation (dMCAO+lt-NES). An overview of the location of graft-derived cells, using the human cell nuclear marker STEM101, is also included in the transplanted group. The white square depicts the area where images were taken and quantification was performed. Scale bar in A, 1 mm. CC: Corpus callosum. LV: Lateral ventricle. (**B**-**C**) Representative confocal images (**B**) and cell quantification (**C**) of OLIG2$^+$ cells and STEM101$^+$ cells in the middle part of the corpus callosum. Scale bar in B, 20 µm. *$p<0.05$ vs. Sham. #$p<0.05$ vs. dMCAO. &$p<0.05$ vs. total number OLIG2$^+$ in dMCAO+lt-NES cells. n=5 animals per group. Data are shown as mean ± SEM.

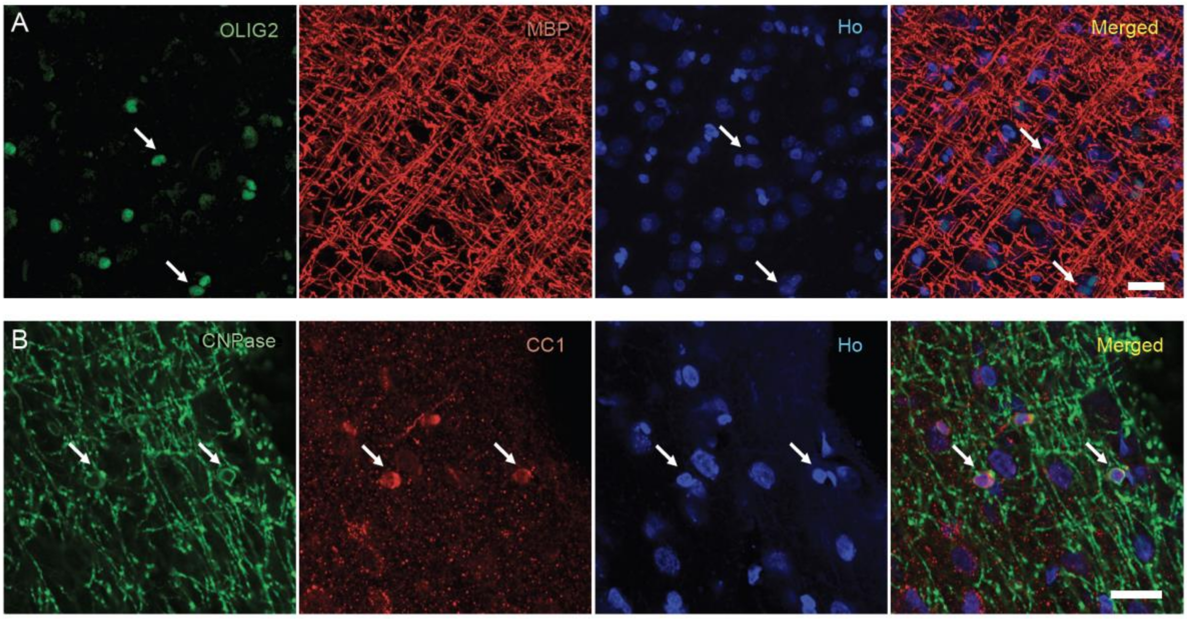

Figure S3. Characterization of the oligodendrocyte population in acute human adult cortical tissue. Related to section: *Grafted human lt-NES cell-derived oligodendrocytes myelinate host axons in adult human cortical tissue.* Representative confocal images of acute human cortical tissue showing the expression of different oligodendrocyte markers: (**A**) the pan-oligodendrocyte marker OLIG2 and myelin basic protein (MBP), and (**B**) a marker for immature and mature oligodendrocytes (CNPase) and mature oligodendrocytes (CC1). Ho, Hoechst. Arrows indicate colocalization. Scale bar, 20 μm.

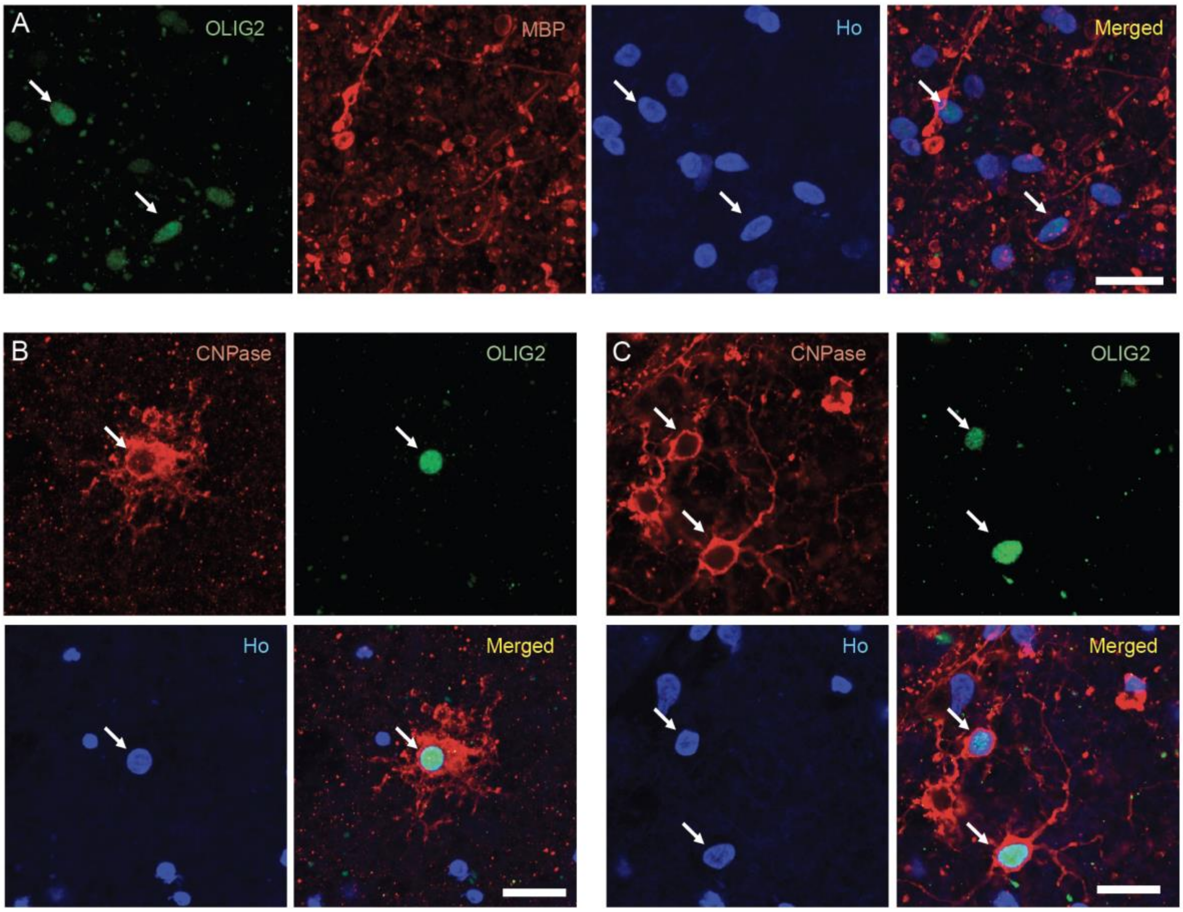

Figure S4. The oligodendrocyte population is preserved in human adult cortical organotypic tissue after 4 weeks in culture. Related to section: *Grafted human lt-NES cell-derived oligodendrocytes myelinate host axons in adult human cortical tissue.* Representative confocal images of 4 weeks-cultured human cortical tissue showing the expression of different oligodendrocyte markers: (**A**) the pan-oligodendrocyte marker OLIG2 and myelin basic protein (MBP); (**B**) a marker for immature and mature oligodendrocytes (CNPase) and OLIG2; and (**C**) a mature oligodendrocyte marker (CC1) and OLIG2. Ho, Hoechst. Arrows indicate colocalization. Scale bar, 20 μm.

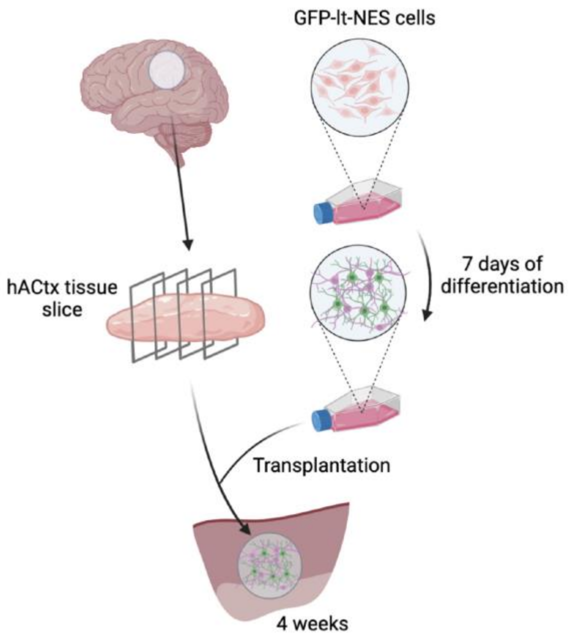

Figure S5. Experimental design of lt-NES cell transplantation onto human adult cortical tissue. Related to section: *Grafted human lt-NES cell-derived oligodendrocytes myelinate host axons in adult human cortical tissue.* Lt-NES cell-derived progenitors differentiated for 7 days are transplanted onto 300 µm slices of human adult cortical tissue cultured for a week. Coculture is maintained for 4 weeks before fixation. Figure generate using Biorender.

Antibody	Host species	*In vitro*	*In vivo*	*Ex vivo*	Notes	Company
Primary Antibodies						
CC1	Mouse	1:200	1:200	1:1000	Antigen retrieval (AR)	Abcam
CNPase	Rabbit	-	1:500	1:500	AR	Abcam
DCX	Goat	1:400	-	-		Santa Cruz
GFP	Chicken	-	-	1:1000		Merck Millipore
MAP2	Chicken	1:500	-	1:1000		Abcam
MBP	Mouse	1:1000	1:500	1:1000	AR	Biolegend
NEUN	Rabbit	-	-	1:1000		Abcam
NEUROFILAMENT	Chicken	1:1000	-	-		Abcam
NG2	Rabbit	-	1:100	-		Merck Millipore
OLIG2	Rabbit	1:500	1:500	1:500	AR	Abcam
SOX10	Goat	1:200	-	1:500	AR	Santa Cruz
STEM101	Mouse	-	1:500	-		Stem Cells
STEM121	Mouse	-	1:500	-		Stem Cells
Secondary Antibodies						
488 anti-Chicken	Donkey	1:500	-	1:500		Jackson ImmunoResearch
488 anti-Goat	Donkey	1:500	-	-		Jackson ImmunoResearch
488 anti-Mouse	Donkey	-	1:500	-		Jackson ImmunoResearch
488 anti-Rabbit	Donkey	1:500	1:500	1:500		Jackson ImmunoResearch
Cy3 anti-Goat	Donkey	1:500	-	1:500		Jackson ImmunoResearch
647 anti-Chicken	Donkey	-	-	1:500		Jackson ImmunoResearch
647 anti-Mouse	Donkey	1:500	1:500	1:500		Jackson ImmunoResearch
647 anti-Rabbit	Donkey	1:500	1:500	1:500		Jackson ImmunoResearch
647 Streptavidin	Donkey	-	-	1:500		Jackson ImmunoResearch

Table S1. List of primary and secondary antibodies. **Related to section: *Immunostainings and quantifications***.

SUPPLEMENTAL EXPERIMENTAL PROCEDURES

Derivation of iPS cell-derived lt-NES cells

Human dermal fibroblasts from healthy adult donors were subjected to Sendai virus transduction with the reprogramming factors Oct4, Sox2, KLF4, and c-MYC (CytoTune iPS 2.0 Sendai Reprogramming kit, Invitrogen). Colonies were picked to establish iPS cell lines using mTeSR medium (Invitrogen). For neural induction, iPS cells were split and colonies were gently resuspended in embryoid body (EB) medium (Dulbecco's modified Eagle medium/F12 [DMEM/F12], 10% KSR, 2-Mercaptoethanol [1:1000], nonessential amino acids [NMEAA] [1:100], Glutamine [1:100]) with Rock Inhibitor (1:1000), 3 μM Dorsomorphin (Sigma-Aldrich) and 10 μM SB431542 (Sigma-Aldrich). On day 5, EBs were collected and plated on poly-L-ornithine/ laminin-coated plates in an EB medium with 3 μM Dorsomorphin and 10 μM SB431542. On day 6, the media was changed to N2 medium (DMEM-F12 [without Hepes + Glutamine], N2 [1:100], Glucose [1.6 g/L]) supplemented with 1 μM Dorsomorphin and 10 ng/mL bFGF. Neural rosettes were carefully picked, six days later, and grown in suspension in an N2 medium with 20 ng/mL bFGF. On day 14, neural rosette spheroids were collected, and the small clumps obtained were grown in adhesion on poly-L-ornithine/laminin-coated dishes in the presence of 10 ng/mL bFGF, 10 ng/mL EGF (both from Peprotech) and B27 (1:1000, Invitrogen). The lt-NES cell line was routinely cultured and expanded on 0.1 mg/mL poly-L-ornithine and 10 mg/mL laminin (both from Sigma)-coated plates into the same media supplemented with FGF, EGF, and B27 and passaged at a ratio of 1:2 to 1:3 every second to the third day using trypsin (Sigma).

Distal middle cerebral artery occlusion and cell transplantation

Animals were housed in individually ventilated cages under standard temperature and humidity conditions and a 12-h light/dark cycle with free access to food and water.

The focal ischemic injury was induced in the somatosensory cortex by distal middle cerebral artery occlusion (dMCAO) as described previously (Chen et al., 1986; Oki et al., 2012). Briefly, Animals were anesthetized with isoflurane (3.0% induction; 1.5% maintenance) mixed with air and the temporal bone was exposed. A craniotomy of 3 mm was made, the dura mater was carefully opened, and the cortical branch of the middle cerebral artery was ligated permanently by suture. Both common carotid arteries were isolated and ligated for 30 min. After releasing common carotid arteries, surgical wounds were closed.

Intracortical transplantation of cortically fated GFP$^+$ lt-NES cell-derived progenitors was performed stereotaxically 48 h after dMCAO as described previously (Palma-Tortosa et al., 2020; Tornero et al., 2013). Briefly, on the day of surgery, cortically primed cells on their third day of differentiation were resuspended to a final concentration of 1 x 10^5 cells/μL in cytocon buffer. A volume of 1 μL was injected at two sites with the following coordinates: anterior/posterior: +1.5 mm; medial/lateral: 1.5 mm; dorsal/ventral: - 2.0 mm; and anterior/posterior: + 0.5 mm; medial/lateral: 1.5 mm; dorsal/ventral: -2,5 mm.

Organotypic cultures of the adult human cortex

The surgically resected tissue was instantly kept in ice-cold modified human artificial cerebrospinal fluid and sliced on a vibratome (Leica VT200S). Slices of 300 μm thickness were kept in a rinsing medium until they were fixed with 4% formaldehyde for acute characterization of the tissue or transferred to cell culture inserts containing Alvetex scaffold membranes (Reinnervate) in 6 well plates filled with human adult cortical media (Brain Phys medium [without phenol red] supplemented with B27 [1:50], glutamax [1:200] and gentamycin [50 mg/mL]), and incubated in 5% CO2 at +37ºC. The organotypic slices were maintained in culture for 1 week before transplantation of GFP$^+$ lt-NES cells and kept 4 weeks more before characterization of grafted cells. Non-transplanted slices maintained for 4 weeks in culture were used for characterization by immunohistochemistry and electrophysiological recordings of the neural populations in cultured tissue.

Immunocytochemistry and Immunohistochemistry

Cortically fated lt-NES cells plated on glass coverslips were fixed at different time points of differentiation in 4% formaldehyde (Sigma) for 20 min at room temperature. Cells were permeabilized with 0.025% Triton X-100 in 0.1 M potassium phosphate buffered saline (KPBS) and blocked with 5% of normal donkey serum (NDS) for 45 min at room temperature. Afterward, primary antibodies (**Table S1**) diluted in blocking solution were applied overnight at +4 ºC followed by 3 rinses with KPBS. Fluorophore-conjugated secondary antibodies (**Table S1**) (1:500, Jackson Laboratories) diluted in blocking solution were applied for 2 h at room temperature. Subsequently, cells were rinsed 3 times and nuclei were stained with Hoechst (Molecular Probes or Jackson Laboratories) for 10 min at room temperature. Stained glass coverslips were mounted on slides with Dabco (Sigma) mounting media.

Regarding immunohistochemistry in rat slices, the stored sections were rinsed 3 times with KPBS and incubated in a blocking solution for 1 h (10% NDS and 0.25 Triton X-100 in 0.1 M KPBS [TKPBS]). The rest of the procedure follows that outlined above for cell cultures. The list of primary and secondary antibodies used can be found in **Table S1.**

For staining in human organotypic cultures, slices were fixed with 4% formaldehyde overnight at + 4 ºC and rinsed 3 times with KPBS for 15 min. Slices were then incubated overnight at + 4 ºC in permeabilization solution (0.02% Bovine serum Albumin [BSA], 1% Triton X in PBS), and overnight at +4 ºC in blocking solution (KPBS, 0.2% Triton X-100, 1% BSA, Sodium azide [NaN3] [1:10000] and 10% NDS). Primary antibodies were diluted in a blocking solution and incubated for 48 h at +4 ºC. Secondary antibodies were then diluted in a blocking solution and applied for 48 h at +4 ºC. Slices were washed 3 times with KPBS and incubated in Hoechst for 2 h at room temperature. Finally, mounted with Dabco after rinsing with deionized water.

Some stainings required antigen retrieval (**Table S1**) before the permeabilization step. Cells, rat sections, or human organotypic cultures were incubated with sodium citrate pH 6.0 Tween 0.05%, for 30 min (for cells and sections) or 2 h (for organotypic cultures) at +65 ºC.

Immuno-electron microscopy

For iEM in rat tissue, frontal 100 µm sections of the whole brain were cut on a Vibratome VT1000A (Leica, Germany). The sections were cryoprotected, freeze-thawed in liquid nitrogen, and incubated overnight in primary goat anti-GFP antibody (1:500, Novus Biologicals) at +4 ºC. The tissue was then incubated at room temperature for 2 h with biotinylated rabbit anti-goat secondary antibody (1:200, DakoCytomation), and avidin-biotin-peroxidase complex (ABC) (Vector Laboratories) followed by 3,3′-diaminobenzidine tetrachloride (DAB) and 0.015% hydrogen peroxide. Following the DAB reaction, sections were processed for iEM. DAB-immunostained sections of rat brain tissue and human organotypic cultures were postfixed in 1% osmium tetroxide in 0.1 M PBS, dehydrated in a graded series of ethanol and propylene oxide, and flat-embedded in Epon. Ultrathin sections were cut with a diamond knife. For post-embedding immunogold labeling of GFP, ultrathin sections were incubated overnight in primary goat anti-GFP antibody (1:500, Novus Biologicals) at +4 ºC. A secondary antibody (donkey anti-rabbit IgG conjugated to 12 nm colloidal gold; Jackson Laboratories) diluted 1:20 in 0.1% BSA in PBS was added for 1.5 h, then washed with PBS. Sections were then fixed with 2% glutaraldehyde, then washed with PBS followed by dH_2O. Sections were stained with uranyl acetate and lead citrate. Ultrathin sections were examined and photographed using a transmission electron microscope JEM- 100CX (JEOL, Japan).

Electrophysiological recordings

For whole-cell patch-clamp recordings, 4 weeks-old human adult cortical organotypic slices were transferred to a recording chamber and were constantly perfused with carbogenated human artificial cerebrospinal fluid (hACSF, in mM: 129 NaCl, 21 $NaHCO_3$, 10 glucose, 3 KCl, 1.25 NaH_2PO_4, 2 $MgSO_4$ and 1.6 $CaCl_2$, pH ~7.4) during the recordings. Recordings were performed with a HEKA EPC10 amplifier using PatchMaster software for data acquisition. The internal pipette solution contained (in mM): 122.5 K-gluconate, 12.5 KCl, 10 HEPES, 2.0 Na_2ATP, 0.3 Na_2-GTP, and 8.0 NaCl). Biocytin (1-3 mg/mL, Biotium) was dissolved in the pipette solution for *post-hoc* identification of recorded cells.

To study the ability to generate AP and its characteristics either a current ramp of 0-300 pA, or 10 pA current steps were applied at resting membrane potential (RMP) in the current clamp configuration. In

voltage clamp mode, sodium and potassium currents were evoked by a series of 10 mV steps ranging from -70 mV to +40 mV. RMP was measured in current clamp mode immediately after establishing the whole-cell configuration. Input resistance (Ri) was calculated from a 10 mV pulse and monitored throughout the experiment. Data were analyzed offline with FitMaster and IgorPro software.

SUPPLEMENTAL REFERENCES

Chen, S.T., Hsu, C.Y., Hogan, E.L., Maricq, H., and Balentine, J.D. (1986). A model of focal ischemic stroke in the rat: reproducible extensive cortical infarction. Stroke *17*, 738-743. 10.1161/01.str.17.4.738.

Oki, K., Tatarishvili, J., Wood, J., Koch, P., Wattananit, S., Mine, Y., Monni, E., Tornero, D., Ahlenius, H., Ladewig, J., et al. (2012). Human-induced pluripotent stem cells form functional neurons and improve recovery after grafting in the stroke-damaged brain. Stem Cells *30*, 1120-1133. 10.1002/stem.1104.

Palma-Tortosa, S., Tornero, D., Gronning Hansen, M., Monni, E., Hajy, M., Kartsivadze, S., Aktay, S., Tsupykov, O., Parmar, M., Deisseroth, K., et al. (2020). Activity in grafted human iPS cell-derived cortical neurons integrated in stroke-injured rat brain regulates motor behavior. Proc Natl Acad Sci U S A *117*, 9094-9100. 10.1073/pnas.2000690117.

Tornero, D., Wattananit, S., Gronning Madsen, M., Koch, P., Wood, J., Tatarishvili, J., Mine, Y., Ge, R., Monni, E., Devaraju, K., et al. (2013). Human induced pluripotent stem cell-derived cortical neurons integrate in stroke-injured cortex and improve functional recovery. Brain *136*, 3561-3577. 10.1093/brain/awt278.
